# Origins of the Superiority of Oscillating Electric Fields for Disrupting Senile Plaques: Insights from the 7-Residue Fragment and the Full-length Aβ-42 Peptide

**DOI:** 10.1021/jacs.4c14791

**Published:** 2025-01-08

**Authors:** Surajit Kalita, David Danovich, Sason Shaik

**Affiliations:** Institute of Chemistry, The Hebrew University of Jerusalem, Edmond J. Safra Campus, Givat Ram, Jerusalem 9190401, Israel

## Abstract

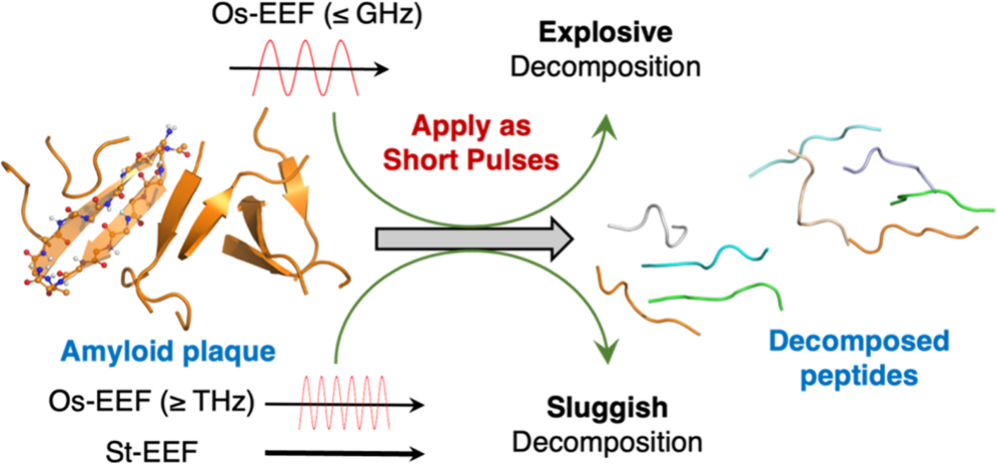

Our recent molecular dynamics simulations of decomposing Alzheimer’s disease plaques, under oscillating- and static external electric fields (Os-EEFs and St-EEFs), revealed the superiority of Os-EEF for decomposing plaques consisting of the 7-residue peptide segment. This conclusion is now reinforced by studying the dimers of the short peptides and trimers of the full-length Aβ-42 peptide. Thus, the dispersed peptides obtained following St-EEF applications reformed the plaques once the St-EEF subsided. In contrast, *plaques originating from the application of Os-EEF remained dispersed for long time scales*. The present study provides insights into these results by modeling the decomposition modes that transpire under both field types. Additionally, this study provides insights into the frequency effects on the decomposition processes within the THz–MHz regions. The simulation shows that the Os-EEF in the lower frequency range (≤GHz) decomposes the plaque on a time scale of ∼50 ns, whereas the higher frequency Os-EEFs (≥THz) are less effective. As such, Os-EEFs with moderate-to-low frequencies (≤GHz) lead to an “explosion,” whereby the peptides fly distantly apart and inhibit plaque reformation. By contrast, St-EEFs form parallel peptide pairs, which are stabilized by the EEF due to the large dipole moment of the ensemble. Thus, St-EEF applications lead to sluggish and reversible plaque decomposition processes. We further conclude that the Os-EEF impact is maximal for short pulses, which prevents the EEF propensity to arrange the peptides in parallel pairs. The superiority of the Os-EEF over the St-EEF is maintained irrespective of the peptides’ length. A model is formulated that predicts the dependence of the decomposition time scale on the EEF.

## 1 Introduction

There has recently been significant progress in our understanding of chemical reactivity, structure modification, and ion separation, under the influence of external electric fields (EEFs).^1−17^ In addition to computational studies,^1−7^ chemists have been testing EEF applications using a variety of systems and methods.^7−17^ Notably, the experimental study of Aragonès et al.^9^ on the EEF catalysis of the Diels−Alder reaction serves as a proof-of-principle for the potential of EEFs to enhance chemical reactivity.

Similarly, Warshel had hypothesized^18−20^ that the extraordinary catalytic power of enzymes originates from electrostatic effects, a hypothesis that has been supported by elegant experimental studies of Boxer’s group.^21,22^ Boxer et al. used vibrational Stark spectroscopy and demonstrated that the enzymatic environment of ketosteroid isomerase can generate an electric field as strong as −144 MV/cm (−1.44 V/Å), which significantly increases the reaction rate.^21^ This discovery further demonstrates that proteins or enzymes may have a significant level of tolerance to strong electric fields.

At the same time, medical research has revealed breakthrough discoveries in EEF applications for treatments of diseases,^23−30^ e.g., applying DBS (deep brain stimulation),^23−25^ which uses St-EEF, and TTFields (tumor-treating fields),^26−30^ which use Os-EEFs for treatments of solid tumors. Apart from these two therapeutic techniques, there are several reported applications of electric fields in biology and medicine,^31^ such as wound healing,^32^ neuronal stimulation,^33^ etc. Indeed, electric fields have emerged as useful tools in a variety of processes.

Similarly, computational methods have been utilized to investigate the behavior of peptide conformations under EEFs, including both static and electromagnetic fields.^34−38^ Understanding the various conformations of peptides at the molecular level is also important from the medical research perspective, as peptide misfolding can lead to several diseases, such as neurodegenerative disorders, e.g., Alzheimer’s^39^ and Parkinson’s^40^ diseases. Nevertheless, there remains a gap in systematic studies that integrate these computational findings with existing medical treatments. As such, in our previous work,^41^ we computationally demonstrated the efficacy of an EEF in decomposing β-amyloid aggregation and attempted to establish a conceptual linkage between such an approach and existing therapeutic modalities, such as TTFields^26−30^ therapy. We further anticipate that our study may expand the scope of investigating the impact of EEFs on other types of aggregates, such as the metabolites of the urea cycle and uric acid pathway.^42^

**Scheme 1:**
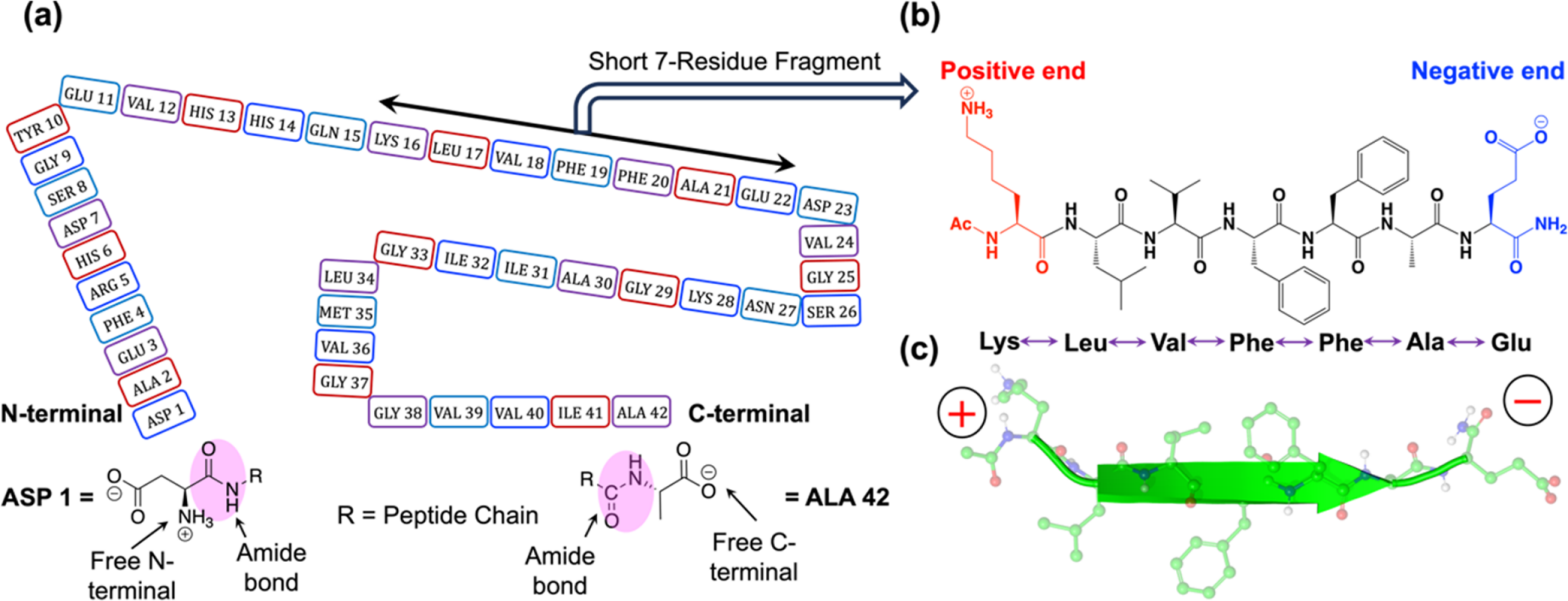
(a) Schematic Representation of the Full-length Aβ-42 Peptide; (b) 7-Residue Peptide (See Ref (49).), Which Serves as a Model of the Behavior of Full-Length Aβ−42 Peptide; (c) Green Arrow-like Cartoon Symbolizes the Peptide, While the Constituent Atoms Are Represented Using a Ball-and-Stick Model: for Clarity, All Nonpolar Hydrogens Are Omitted

In our previous molecular dynamics (MD) simulations of a senile plaque model,^41^ which involves 10 peptides, each consisting of 7 residues (see Scheme 1), we found that the St-EEF (0.02 V/Å) initially changes the plaque into a structure of organized peptide pairs (OPP). This new structure, which possesses a substantial dipole moment of 1131 D, gains significant stability due to the interaction between its huge dipole and the St-EEF. This was followed by a sluggish process of separating the peptide pairs and finally decomposing the plaque into 10 individual peptides on a time scale of ∼1200 ns.

By contrast to St-EEF, the Os-EEF of the same intensity (0.02 V/Å) decomposes the plaque readily and effectively at a time scale of 50 ns, at the GHz frequencies (0.1−1 GHz). *This superiority of Os-EEF is supported by the in vitro studies* of disrupting amyloid aggregation by Saikia et al.,^43^ the fibrillar disruption in human serum albumin by Sen et al.,^44^ and the theoretical study by Vargas-Rosales et al.^38^ of Os-EEF effects on a β-amyloid dimer, generated from the AlphaFold-multimer.^45^

In conducting computational mechanistic studies and linking our findings with established noninvasive TTFields therapy, we fully admit that the field intensity of 0.02 V/Å employed by us is significantly higher than the clinically used EEFs. *Nevertheless, we utilize this intensity as a model because it allows us to perform MD simulations for reasonably long time scales and provides meaningful mechanistic insights.*

However, one might argue that peptides may not exhibit similar behavior when the field strength is reduced to clinically relevant levels. Nevertheless, this concern is not supported by the experimental evidence. Thus, for example, Saikia et al.^43^ demonstrated in vitro disruption of amyloid aggregation at a field strength of 3 × 10^−6^ V/Å brings about significant changes within approximately 24 h. Similarly, the noninvasive TTFields therapy^26−30^*is recommended for continuous use* (no less than 18 h per day) over several months to enhance survival chances.^46^ Thus, it is seen that biological systems, whether amyloid aggregates or tumor cells require a long time to respond to low-intensity EEF. At this point, we note that Saikia et al.^43^ applied a similar field strength to another β−sheet-containing enzyme, trypsin, and found that trypsin did not lose its functional activity, indicating that EEF can be selectively employed to disrupt amyloid aggregates.

Similarly, computational work by Vargas-Rosales et al. showed that decreasing EEF strength slows the decay of β-sheet structures in dimeric Aβ−42 peptides.^38^ Additionally, English et al. demonstrate that the observation of tangible field effects at lower field strengths requires rather long simulation time scales.^47,48^

As shown later (e.g., Figure 12), our theoretical results with EEFs of 0.02−0.01 V/Å enabled us to *derive an equation that predicts the dependence of the time scale on the used EEF, all the way to EEFs equal to or smaller than 10*^−6^*V/Å*. As such, the present computational study reveals a trend similar to those found by experimental methods. Thus, although there is no direct correlation between the simulation time scale and actual time duration, it is observed that as the field strength decreases, both the theoretical time scale and the experimental duration increase.

The major aim of the present manuscript is to understand the underlying root cause for the superiority of Os-EEF over St-EEF, as discovered in our previous work.^41^ Additionally, it is still unclear how changes in the frequency of the Os-EEF affect the effectiveness of plaque decomposition. Therefore, this study involves the following four-pronged mechanistic explorations.

(a) An important goal of this manuscript is to derive a general model that links the simulation time scale to the magnitude of the applied EEF, thus enabling one to predict experimental trends (see, e.g., Figure 12 later).
(b) Comprehending effects of the Os-EEF frequency range. This is achieved by both lowering the frequency to the MHz range and increasing it to the THz range. As discussed by English et al.,^47,48^ there are some practical computational limitations when studying very low frequencies; therefore, we restricted our simulations to frequencies higher than or equal to 20 MHz.
(c) Augmentation of the MD simulations by comparative analyses of the decomposition mechanisms of the most elementary structure, the head-to-tail (HT) dimer, under both St-EEF and Os-EEF.
(d) Using a trimer of the full-length Aβ-42 peptide for validating the superiority of Os-EEF in the decomposition process.

As will be demonstrated, our findings reveal that the Os-EEF with moderate-to-lower frequencies (≤GHz) requires a significantly smaller simulation time scale than its static counterpart. By contrast, at high frequencies (≥THz), *the Os-EEF behaves similarly to the St-EEF*. We note that a short decomposition time scale means that the peptides do not explore much of the potential energy surface, thus likening the process to an **“explosion**.**”** Quantitative analysis shows that in the lower frequency range (≤GHz), the HT dimer decomposes at a higher velocity and over greater distances compared to the St-EEF. Finally, *we demonstrate that a similar mechanism applies in the case of the full-length Aβ-42 trimer.*

With all of the above features, our study unifies the mechanistic roles of the Os-EEF vs St-EEF in the plaque decomposition process and provides the hitherto missing mechanistic understanding for the occurrence of ‘**plaque explosion**’ under the Os-EEF as opposed to the sluggish and reversible decomposition in the St-EEF.

## 2 Computational Methods

### 2.1 Choice of Model Systems

This study considers two different aggregates: one is a 10-peptide plaque^41^ formed by a short 7-residue segment (see Scheme 1b,c) of the full-length Aβ-42 peptide (see Scheme 1a), and the other is a trimer of the full Aβ-42 peptide. Below, we explain the basis for our choice of these model systems.

The short peptide, first derived by the Tycko group,^49^ consists of residues 16 to 22 and contains crucial hydrophobic residues, which are essential for initiating and inhibiting Aβ-42 amyloid plaque formation.^50−52^ Due to its significant role, ability to form ordered fibrils in water, and computational advantages, it has become a choice model for studying the mechanistic aspects of amyloid plaques,^53−59^ which are exhibited by the full-length Aβ-42 plaques. Furthermore, a recent study by Devi and Paul illustrates that interactions with foreign substances (molecular tweezer) disrupt short 7-residue peptide aggregates in a manner similar to that observed in full-length Aβ−42 peptides, thus further supporting the relevance of short peptides-plaques.^55^ Moreover, the experimental study by Saikia et al.^43^ emphasizes the significance of this short peptide model and obtains results similar to those observed with the full-length Aβ-42 peptide in vitro. Similarly, using this same short peptide, we previously^41^ demonstrated that the Os-EEF is superior to the St-EEF for the disintegration of amyloid plaques. The current study aims to explain this observed superiority.

As extensively reviewed by Ilie et al.,^60^ many previous studies have sought to understand the various mechanistic aspects associated with full-length Aβ−42 by mostly focusing on the monomer and dimer forms. The present study examines the decomposition mechanism of an Aβ−42 trimer, derived from its fibril structure as determined by cryo-electron microscopy,^61^ in the presence of Os-EEF and St-EEF. While using classical MD simulations for investigating the impact of EEF on the Aβ−42 trimer, we note that the EEF can also influence the redox behavior of amino acid residues. However, exploring such events is beyond the scope of classical MD simulations.

### 2.2 General Descriptions of the Simulations

The initial coordinates for the short peptide plaques, along with the surrounding water box, were taken from our previous study.^41^ On the other hand, the Aβ-42 trimer was obtained from PDB id 5OQV,^61^ and a detailed description of the system preparation can be found in Section S.1 and Figure S1 of the SI.

Thereafter, we performed MD simulations in the presence of an Os-EEF of strength 0.02 V/Å using three different frequencies: 20 MHz, 1 THz, and 10 THz. For the Aβ-42 trimer, we also performed simulations with No-EEF and St-EEF. Each case was replicated to confirm our observations.

In consistency with the previous work,^41^ we performed here all of the simulations using the GPU version of AMBER22 software^62,63^ and the ff14SB^64^ and TIP3P^65^ force fields for peptides and water molecules, respectively. A detailed description of various simulation parameters can be found in the SI (see Section S.2). VMD^66^ and PYMOL^67^ software packages were used for visualizing and analyzing MD trajectories.

### 2.3 Choice of EEF Strengths

The strength of the EEF used in this work was tested in our previous study,^41^ which demonstrated that an EEF smaller than 0.02 V/Å (i.e., 0.01 V/Å) also decomposes amyloid plaques. At the same time, the lower EEF requires a longer simulation time scale. As such, we conducted several simulations under weaker electric fields of 0.0175, 0.015, 0.0125, and 0.01 V/Å at a frequency of 0.1 GHz. Remarkably, we observed similar outcomes in comparison to those obtained under the strongest field (0.02 V/Å) condition, albeit with a significantly longer simulation time scale (see Section S.3 and Figures S2−S4). This in turn indicates that the general mechanism of plaque explosion under the Os-EEF will remain the same, regardless of the field strength used. A brief discussion is provided below in Section 3.7, while a more detailed discussion is relegated to the SI (see Section S.4 and Figures S5−S6)

### 2.4 Heating Effects

As reviewed by English et al.,^47,48^ the effects of the EEF can raise the system’s temperature, leading to heating due to field-induced molecular friction or the rotation and translation of atoms. Therefore, the application of an appropriate thermostat is always recommended for all EEF-induced nonequilibrium molecular dynamics (NEMD) simulations unless the system is strictly adiabatic. Accordingly, during all simulations, we employed the Langevin thermostat and found that the EEFs used here do not induce more heating than the thermostat can manage (see Section S.5 and Figure S7 in the SI for a more detailed discussion). This indicates that the system does not overheat when the field strength of 0.02 V/Å is applied.

### 2.5 Entropy Calculations

We conducted entropy calculations of the plaque at various time scales during decomposition, by using the inbuilt normal-mode analysis in AMBER22.^62,63^ For each calculation, we selected six alternative frames around the chosen time scale and considered an average of the six frames. After verifying that the six frames behave similarly to the calculation of the 100 frames, we conducted the remaining calculations using six frames. This choice reduced the heavy computational cost of a larger number of frames.

We further generated four additional entropy data sets, resulting in a total of five data sets, each corresponding to an equivalent number but different frames around the given time scale. First, for each data set, we calculated the change in absolute entropy relative to its initial value. Then, we calculated the standard error (SE) by considering the computed relative entropy values for all 5 data sets for the particular time scale. The SE for the initial plaque was calculated from the absolute entropy. Here, the SE is defined as the standard deviation (SD) divided by the square root of the total number of data sets.

The entropy resulting from the normal-mode analysis employs the harmonic approximation and is contributed by translational, rotational, and vibrational modes. Moreover, we found that for the studied 10-peptide plaque, the change in entropy is predominantly governed by the sum of translational and rotational contributions. Consequently, the entropy change that attends the application of Os-EEF is attributed to the plaque’s decomposition phenomenon and is referred to **as dispersion entropy.**

### 2.6 Description of the Head-to-Tail (HT) Dimers

The umbrella sampling method^68^ was used to calculate the potential of mean force (PMF) for the separation of the HT dimers into separated peptides. In so doing, we used the center of mass (COM) restraint procedure (detailed methods and specific parameters outlined in the SI: Section S.6 and Figure S8). Accordingly, the separation coordinate we used is the distance between the COMs of the two peptides in the HT dimer. The PMF was computed based on the COMs distances.

### 2.7 Quantitative Aspects of the Decomposition of the HT Dimer

We determined the rate of the HT dimer decompositions in the presence of the Os-EEF and St-EEF using an in-house Python code (see page S22 in the SI). The distance between the centers of masses (COMs) of individual peptides in the HT dimer served as the displacement vector.

Employing the secondary structure analysis, DSSP method, incorporated in AMBER22,^62,63^ we identified the frame at which the antiparallel β-sheet lost its entire secondary structure characteristics. Subsequently, we calculated the change in the COM-COM distances from the initial conformation to the point where the HT dimers lost their β−sheet characteristics.

Finally, utilizing the MD trajectory data, we determined the required decomposition times, velocities, and distances of separation during the β−sheet decomposition.

## 3 Results and Discussion

### 3.1 Plaque Explosion Under Os-EEF

Our MD findings are clear-cut. The 10-peptide plaque (see Scheme 1b,c) decomposes at the ∼50 ns time scale in the presence of the Os-EEF when we used a moderate-to-lower frequency (≤GHz) and an EEF strength of 0.02 V/Å. We define this phenomenon as **“plaque explosion”** because the plaque quickly disintegrates into individual peptides *without significantly exploring the potential energy surface*. By contrast, when the frequency is increased to the THz range, we do not observe the “plaque explosion.” The following subsections systematically discuss the results obtained across the different frequencies.

### 3.1.1 Decomposition at the GHz Frequency

Our previous work^41^ provided a detailed discussion on the plaques’ decomposition induced by GHz frequencies (0.1−1 GHz). In this paper, we present results for 0.1 GHz or 100 MHz.

**Figure 1:**
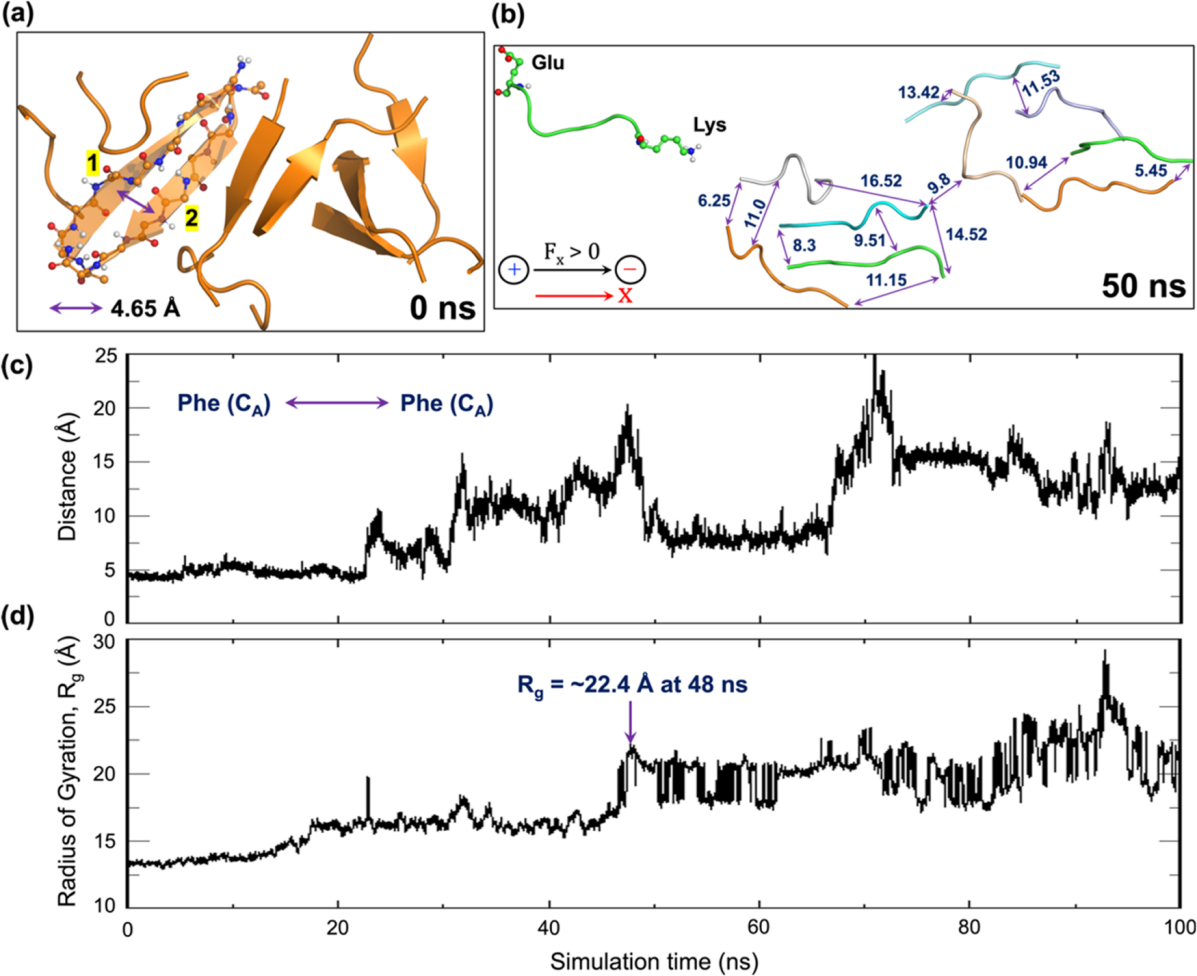
(a) Plaque of the HT dimers generated by the 10 short analogues of the *A*β−42 peptides in the absence of EEF. The double-headed arrow at the bottom left-hand side of the figure specifies the initial distance (4.65 Å) between peptides **1** and **2**. (b) Under an Os-EEF of 0.1 GHz frequency (along X) and EEF = 0.02 V/Å, the plaque disintegrates into separate peptides, at a 50 ns time scale. Representative interpeptide distances are shown in Å units by double-headed arrows between the various dispersed peptides. (c) Illustration of the evolution of interpeptide distance (in Å) with the change of simulation time scale (in ns), focusing on the backbone carbon atoms of two phenylalanine residues: one located in peptide **1** and the other in peptide **2** (initial distance is 4.65 Å, see Figure 1a). (d) Evolution of the radius of gyration (*R*_g_) of peptides over the course of the simulation. The *R*_g_ value at 48 ns is ∼22.4, which is a maximum within the initial 50 ns.

As seen in Figure 1a, in the absence of EEF, β-amyloid peptides are antiparallelly organized in a cross β-sheet fashion, which involves head-to-tail (HT) dimers and which marks the formation onset of larger plaques. By contrast, as shown in Figure 1b, upon the application of the Os-EEF, the closely residing β-amyloid peptides separate significantly and evolve quickly to a decomposed plaque, wherein the peptides are significantly farther away compared with the initial distance of 4.65 Å (see Figure 1a). The substantial peptide separation is also evident from Figure 1c, which plots the evolution of the distance between the backbone atoms of peptides **1** and **2** (see Figure 1a). Thus, the two peptides begin to separate after the initial 20 ns and reach interpeptide distances up to 20 Å at the ∼50 ns time scale. Such an increase in interpeptide distances can also be statistically observed from increasing radius of gyration (*R*_g_) values, as shown in Figure 1d. The increasing *R*_g_ typically reflects a decrease in the compactness of the aggregate.

Additional features can be found in the SI (see Section S.7, Figures S9 and S10). These include representations of plaque decomposition, along with the root-mean-square deviations (RMSD) of the peptide backbone atoms, the evolution of the number of hydrogen bonds, and the decay of the secondary structure over time. Clearly, therefore, Figures 1 and two SI Figures (Figures S9 and S10) demonstrate that the 10-peptide plaque decomposes at a short time scale, wherein the individual peptides disperse over long distances and maintain this well-separated conformation throughout the duration of the simulation. *Thus, the Os-EEF induced instant plaque decomposition!*

### 3.1.2 Decomposition at the MHz Frequency

Next, we tested the impact of reducing the frequency of the Os-EEF to 20 MHz, considering that the selected Os-EEF completes a full oscillation within the decomposition time scale.

**Figure 2:**
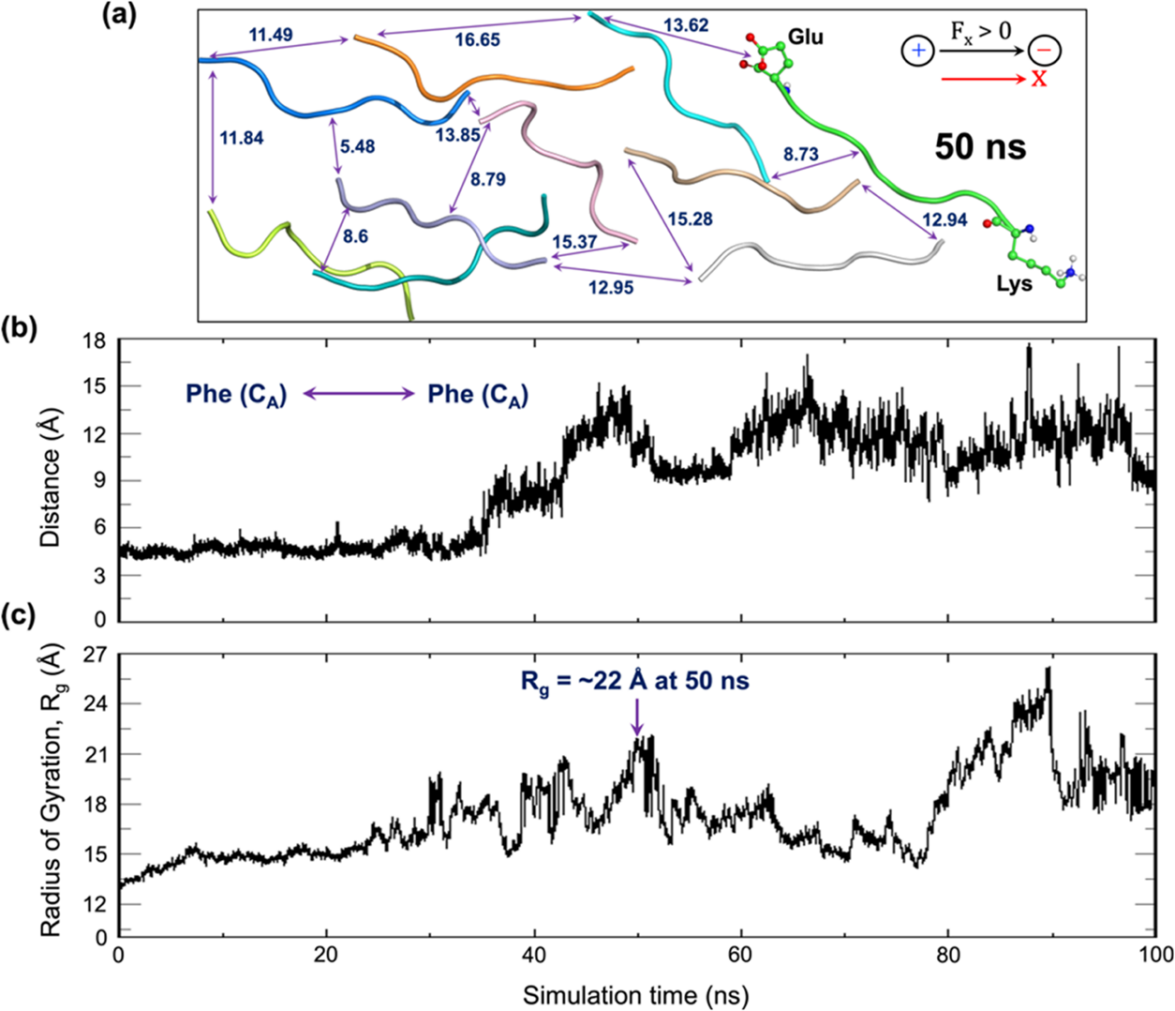
(a) Decomposition of the 10-peptide plaque occurs within a time scale of 50 ns due to exposure to an Os-EEF (along X) of 0.02 V/Å with a frequency of 20 MHz. The double-headed arrows indicate a few representative interpeptide distances (Å) among the dispersed peptides. The black arrow at the top right marks the direction of the applied Os-EEF. (b) Evolution of interpeptide distance (in Å) over the simulation time scale (in ns), focusing on the backbone carbon atoms of two phenylalanine residues—one in peptide 1 and the other in peptide 2, as referred to in Figure 1a (initial distance is 4.65 Å). (c) Evolution of the radius of gyration of peptides with the progress of the simulation. The maximum *R*_g_ value is ∼22 at 50 ns.

A comprehensive description of plaque decomposition at 20 MHz frequency, coupled with an EEF strength of 0.02 V/Å, can be seen in Figure 2. During the 50 ns time scale, we observe a disappearance of the secondary β-sheet, which is *a crucial marker of plaque formation* (see Figure 2a). As shown in Figure 2a, the representative interpeptide distances are longer than the average initial distance of 4.65 Å (Figure 1a). Additionally, Figure 2b clearly illustrates how the interpeptide distance evolves with the simulation progress. Such increasing interpeptide separations can further be corroborated by the increasing values of *R*_g_ throughout the simulation, as shown in Figure 2c.

Furthermore, the decreasing number of hydrogen bonds, from ∼30 at the start of the simulation to around 10−12 at the ∼50 ns time scale, reflects the occurrence of plaque decomposition (see Figure S13). A similar finding was observed in the replica simulations, for which the evolution of *R*_g_, hydrogen bonding and distance over the simulation time reflect the same trend (see Figure S14 and compare to Figure S15 for frequency <20 MHz).

Thus, the results of the Os-EEF at 20 MHz clearly demonstrate that the individually separated peptides are dispersed over long distances (see Figures 2, S13, and S14 in the SI) within the given simulation time scale, indicating the genuine onset of plaque explosion. This is unlike the case of St-EEF, which induces a sluggish decomposition with a long time scale.^41^

### 3.1.3 Decomposition at the THz Frequency

**Figure 3:**
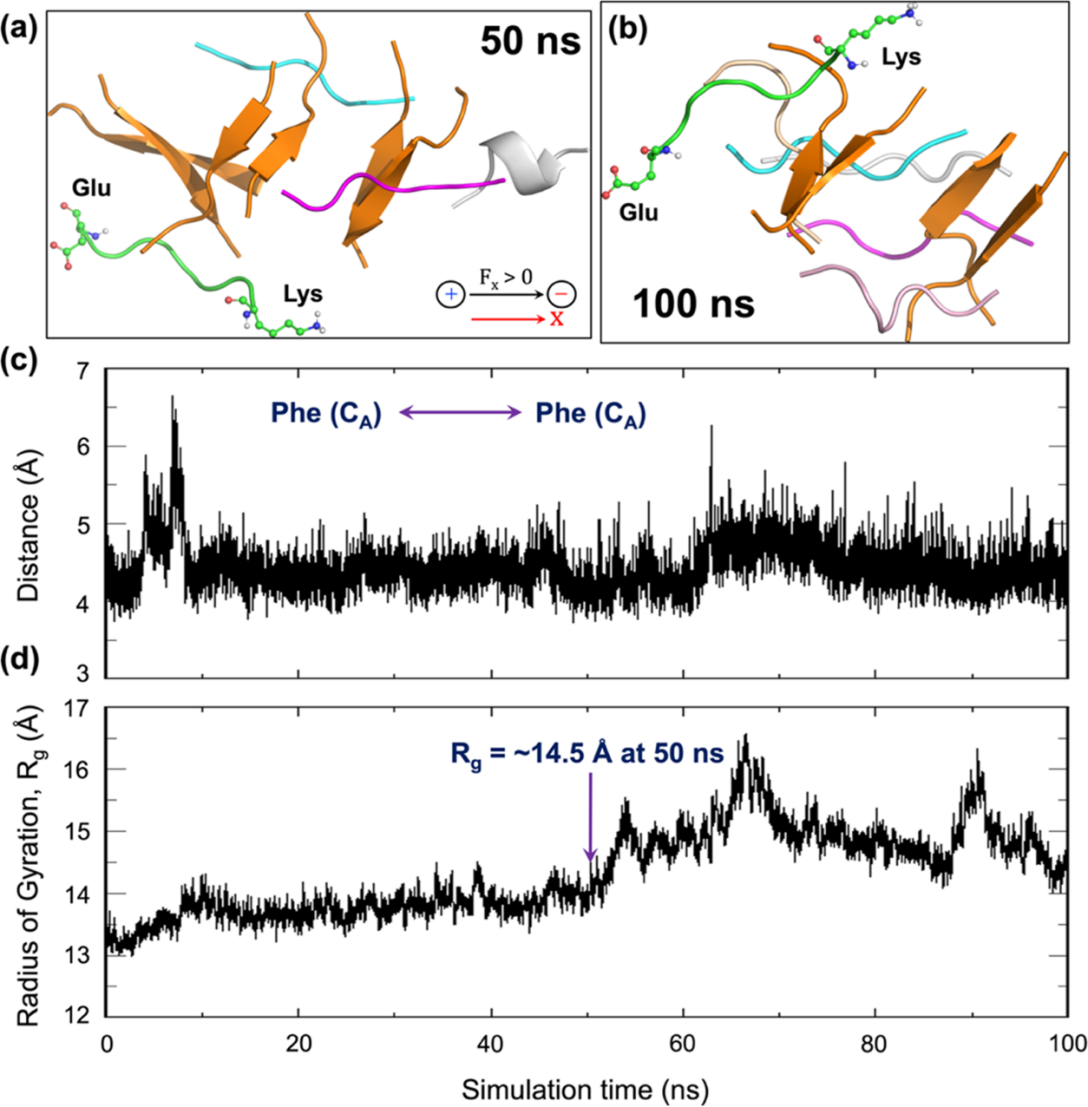
Observed conformational changes of the 10-peptide plaque upon exposure to the Os-EEF (along X) with a 1 THz frequency, with the EEF strength of 0.02 V/Å, at (a) the 50 ns and (b) the 100 ns time scale. The black arrow at the bottom of (b) marks the direction of the applied Os-EEF. (c) Evolution of interpeptide distance (in Å) over the simulation time scale (in ns), focusing on the backbone carbon atoms of two phenylalanine residues—one in peptide 1 and the other in peptide 2, as referred to in Figure 1a (initial distance is 4.65 Å). (d) Evolution of the radius of gyration (*R*_g_) of plaques as the simulation progresses.

To test the effect of high frequency on plaque decomposition, we increased the frequency to 1 THz while keeping the EEF strength the same as before at 0.02 V/Å. As shown in Figure 3a, we did not observe the “plaque explosion” at 50 ns time scale. We then extended the simulation to 100 ns to further monitor the decomposition process; however, as shown in Figure 3b, we did not observe any substantial differences.

The visual observation is further supported by the evolution of the interpeptide distance plot (see Figure 3c), which remains close to its initial value of 4.65 Å (see Figure 1a), whereas the identical peptides separate to 15 to 20 Å in the case of GHz (see Figure 1c) and MHz (see Figure 2b) frequencies. In addition, comparing the *R*_g_ values (see Figures 1d, 2c, and 3d) reveals that at 50 ns, *R*_g_ is approximately 22.4 Å for GHz frequencies, 22 Å for MHz frequencies, and 14.5 for THz frequencies. The findings presented here show that plaques do not efficiently decompose at THz frequencies.

The evolution of hydrogen bonding, which is approximately 10−12 for GHz and MHz (see Figures S9b and S13) compared to 18−20 for THz (see Figure S16), also highlights the smaller effectiveness of the THz frequency in decomposing the plaque. A replica simulation was performed to observe the repeatability of the results, as shown in Figure S17.

To generalize our observations at high frequencies, we increased the frequency to 10 THz and conducted an additional simulation (see Figure S18), which also revealed that plaques do not exhibit “explosion” at this frequency.

### 3.2 Making Sense of the Plaque Explosion Under Os-EEF

In the following subsections, we attempt to comprehend the mechanism of “plaque explosion” and the fate of individual peptides post decomposition. Note that, in the following sections, whenever we refer to the Os-EEF, the respective frequency is moderate to low (≤GHz), unless explicitly specified otherwise.

### 3.2.1 Elucidation of the Mechanisms of Plaque Explosion

**Figure 4:**
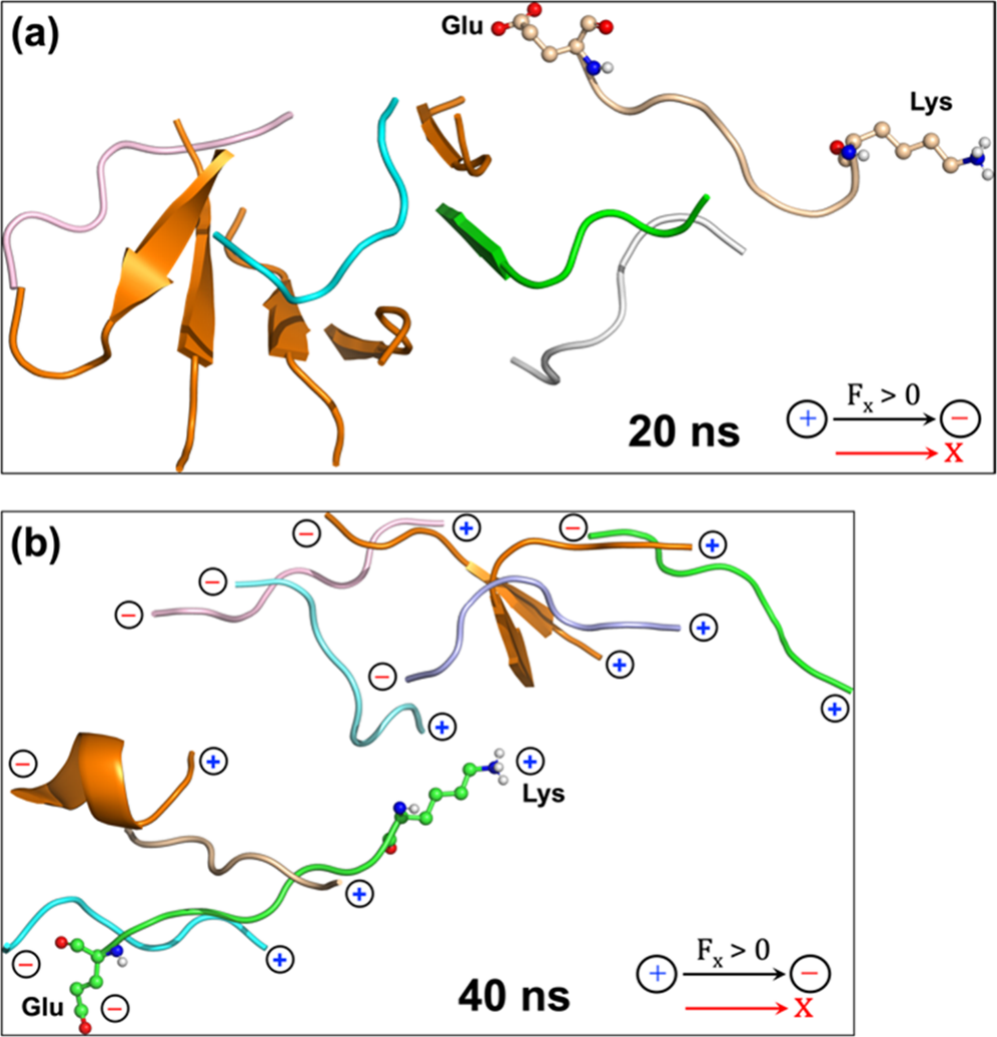
Conformational changes observed during the exposure of the plaque to an Os-EEF (the EEF is in the X direction) with a strength of 0.02 V/Å at a frequency of 0.1 GHz at (a) 20 ns and (b) 40 ns. The positive and negative signs in panel (b), on the peptide ends, represent, respectively, the positively charged lysine and negatively charged glutamate for each peptide.

To extract the requisite insight, we closely monitored the initial 50 ns MD trajectory. Thus, we observed that, **due to the phase change, as well as the effective magnitude and varying nature of the Os-EEF**, the plaque begins to disintegrate very quickly and without much of a motion exploration at ∼20 ns, as shown in Figure 4a. As such, during the 20 ns time scale, the plaque effectively begins to lose its secondary β-sheet content and forms individually separated peptides.

Subsequently, as the simulation progresses, the individually separated peptides attempt to align along the direction of the applied Os-EEF due to their high individual dipole moment (125 D each).^41^ As shown in Figure 4b, a conformation is formed that can be termed as random parallel pairs (**RPP**). The alignment of peptide pairs with the applied Os-EEF can be seen by examining the locations of positive and negative charges in Figure 4b. However, the **RPP** conformation maintains long distances between the individual peptides in each pair and is unstable in the presence of the Os-EEF due to the change of the phase and effective magnitude of the field. As such, the **RPP** structure decomposes after a 40 ns time scale (see Figure 4b) to the individual peptides, which are randomized in the field (see also Figures 1b and 2a above).

### 3.2.2 Quantitative Interpretation of the Decomposition Mechanism for a Frequency of 0.1 GHz

**Figure 5:**
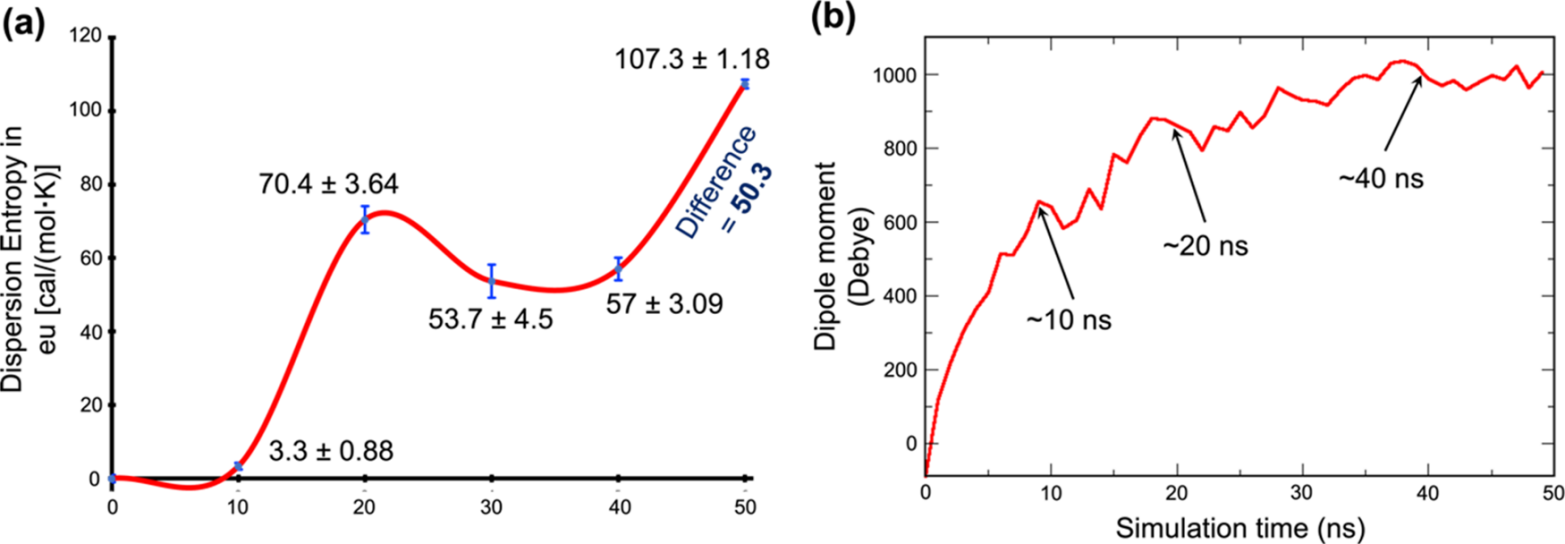
(a) Changes in the dispersion entropy of the plaque, calculated at intervals of 10 ns simulation time scale. All values are in units of eu [cal/(mol·K)] and are relative to the entropy at 0 ns. Error values denote the standard error (S.E.). At time *t* = 0, the SE of absolute entropy is ±0.98. (b) Evolution of the resultant dipole moment of the plaque with the progress of the simulation in the presence of the Os-EEF with a strength of 0.02 V/Å and frequency of 0.1 GHz.

Figure 5 shows the changes of the dispersion entropy and dipole moment of the plaque over a time scale in an Os-EEF of 0.1 GHz. These changes, which are not monotonous, reflect the nature of the mechanistic event. Thus, during the first 10 ns of the simulation in Figure 5a, there is a negligible change in entropy, which is attended by a sharp rise in the dipole moment to approximately 650 D at around 10 ns in Figure 5b. This rise in the dipole moment, along with the subtle change in dispersion entropy, mirrors the reorientation of the ensemble of 10 peptides along the direction of the EEF, without the occurrence of plaque decomposition.

However, the drastic jump in entropy (3.3 → 70.4 eu) in Figure 5a, which occurs between 10 to 20 ns, clearly indicates an increase in the randomness of the plaque. This change is caused by the disruption of the plaque to form well-separated peptides (Figure 4a). These peptides then attempt to align along the direction of the applied field, resulting in a further increase in the dipole moment to around 870 D (see Figure 5b).

Interestingly, the entropy drops by 16.7 eu after 20 ns and remains nearly constant up to 40 ns (Figure 5a). This drop is attributed to the stabilization of separated peptides through their individual dipole interaction with the applied field, leading to a decrease in overall randomization. Consequently, this field-dipole interaction brings about the formation of the **RPP** conformation (see Figure 4b) and contributes thereby to a gradual increase in the dipole moment value from 870 to ∼1000 D or more (see Figure 5b).

After 40 ns, a substantial rise in entropy, ca. 50.3 eu, is observed, indicating further randomization events in the plaque under the Os-EEF (see Figure 5a). This change reveals that the **RPP** conformations get quickly destabilized, leading to the formation of individual peptides and mark the occurrence of “plaque explosion.” Furthermore, around the 40 ns time scale, a slight drop in the overall dipole moment is observed, reaching ∼960 D, suggesting a transition to a more random conformation of peptides. (see Figure 5b).

In summary, therefore, the mechanism of plaque explosion evolves in steps. First, the plaque rapidly disintegrates under the Os-EEF (of 0.1 GHz), giving rise to individually separated peptides. These peptides align along the applied field, forming a conformation labeled as **RPP**. In the second step, the so-formed **RPP** conformation undergoes destabilization due to the phase and effective magnitude alterations of the Os-EEF that disperses the system and results in “plaque explosion.”

A similar mechanistic conclusion can also be drawn from the percentage of β-sheets decay in the presence of Os-EEF. As depicted in Figure S19, there is a sharp reduction in β-sheet content after 20 ns, which then remains relatively constant from 25 to 45 ns, indicating the formation of the RPP conformation. And finally, an explosion occurs around 50 ns time scale.

### 3.2.3 Fate of Disrupted Peptides after Plaque Explosion in Os-EEF

The above mechanistic discussion naturally raises an intriguing question: What will happen to the individually separated peptide ensembles if we expose them to the Os-EEF for a longer duration?

**Figure 6:**
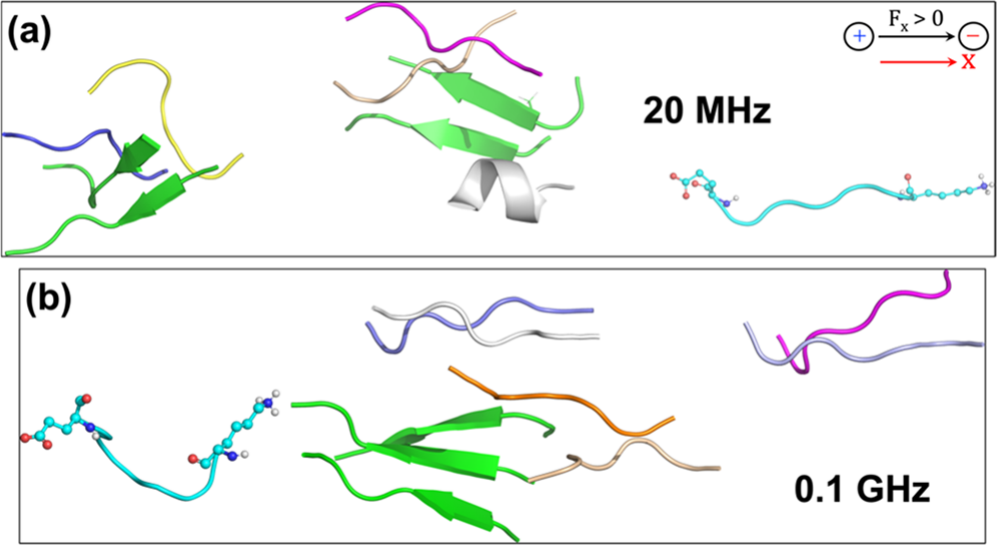
Conformations that demonstrate the slow and incomplete formation of parallel pairs (**PP**) arising from the initially dispersed peptides after prolonged exposure to the Os-EEF (of 0.02 V/Å) at frequencies of (a) 20 MHz and (b) 0.1 GHz.

To answer the question, we extended the Os-EEF simulation to 500 ns, as shown in Figure 6 for the frequencies of 20 MHz and 0.1 GHz. It is seen that prolonging the simulation beyond the time scale of the explosion causes the separated peptides to start building parallel pairs (**PP**). Since a **PP** has a large dipole moment, which gets stabilized by the EEF, the EEF takes over and causes the construction of **PP**s. This observation necessarily suggests that, for efficient decomposition of senile plaques, **it is recommended to restrict the application of Os-EEF to short pulses**. The short duration will, on the one hand, decompose the plaque and, on the other hand, prevent the buildup of **PP**s by the EEF.

Furthermore, one can also anticipate that with prolonged exposure to the Os-EEF, this **PP** arrangement will be disrupted again to **RPP** by the alternating phase of the Os-EEF, leading thereby to a periodic construction–destruction of parallel pairs. Due to computational limitations, a comprehensive test of this idea for the entire plaque was not feasible. Nevertheless, we verified this idea by using a single **PP** pair from Figure 6b. We followed its dynamics by extending the simulation by an additional 300 ns, which verified our idea of periodic construction–destruction of the **PP** arrangement (see Section S.8. and Figures S11 and S12 in the SI for details).

### 3.3 Plaque Explosion during the Application of St-EEF

**Figure 7:**
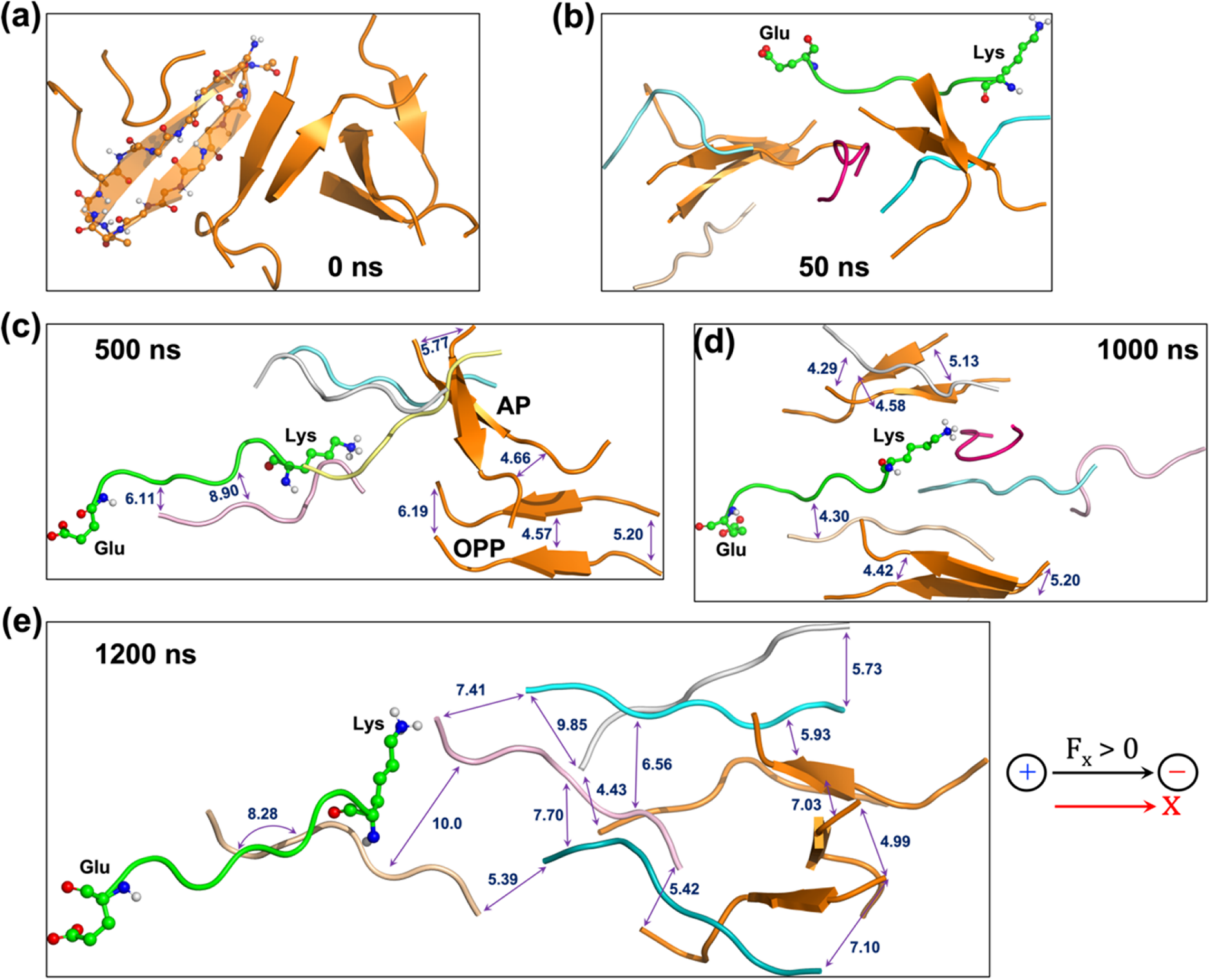
Peptide conformations in the presence of St-EEF (along X) at different time scales: (a) 0 ns, (b) 50 ns, (c) 500 ns, (d) 1000 ns, and (e) 1200 ns. The double-headed arrows show the representative interpeptide distances within the sparse parallel pairs at 500 and 1000 ns (where **OPP** begins) and in the decomposed plaque at 1200 ns. All distances are in Å.

By contrast to Os-EEF, we recall that with St-EEF,^41^ the plaque is transformed into an extended ensemble of organized parallel pairs (**OPP**). This is shown in Figure 7, which summarizes the previous results.^41^

Let us briefly describe the sequence of events in Figure 7. By comparison to the 50 ns decomposition of the plaque under the Os-EEF (see Figure 1b and 2a), the application of the St-EEF, in Figure 7a,7b, does not reveal any distinct conformational change in the plaque at 50 ns. However, a reorientation of the plaque is induced by the interaction of the resultant dipole (166 D)^41^ of the plaque with the applied field. A noticeable conformational change becomes apparent only around 500 ns in Figure 7c, where the plaque begins to disintegrate into individually separated peptides, losing its initial antiparallel β-sheet structure in Figure 7a. The conformation of the ensemble, around 500 ns (see Figure 7c), may be considered a transitional structure, in which almost all antiparallel β-sheets have disintegrated, except for one pair marked as **AP** and another marked as organized parallel pairs (**OPP**); the latter is a newly formed parallel pair.

As depicted in Figure 7c, the process of forming the **OPP** begins at around 500 ns and continues until around 1000 ns (see Figure 7d), where distinct parallel pairs become evident. Subsequently, after a significant exploration of the conformational space, the plaque eventually decomposes at around 1200 ns (Figure 7e), displaying a clear separation among the individual peptides, similar to the 50 ns event in the Os-EEF case (Figures 1b and 2a). Moreover, the plot of the radius of gyration, corresponding to the MD trajectory, is shown in Figure S20, supporting the description provided above.

However, as previously shown,^41^ this separation process is reversible. Thus, once the field subsides, the 10 peptides revert, within the same time scale, to a structure of head-to-tail (HT) dimers. Clearly, therefore, unlike the case for the Os-EEF, which brings about a plaque explosion within a time scale of 50 ns, the St-EEF does not exhibit such a discrete event. The respective decomposition process drags on sluggishly under the influence of the St-EEF to form a set of **OPP** dimers (see Figure 7d), and finally, the plaque decomposes.

### 3.4 Post-Decomposition Events under St-EEF vs Os-EEF and Their Origins

Figure 7 reveals that the interpeptide distances obtained by the application of St-EEF are significantly shorter than those obtained during the Os-EEF (see Figures 1b and 2a). For instance, the organized parallel pairs (**OPP**) formed by St-EEF at 500 and 1000 ns (see Figure 7c,d) exhibit peptide separations, on average 4.5−6 Å, which are much shorter than those observed with Os-EEF at 50 ns; 9−10 Å or higher. (see Figures 1b and 2a). Furthermore, this difference persists even after extending the simulation with St-EEF to 1200 ns (average value of 6−7 Å in Figure 7e). This shorter interpeptide separation in the decomposed state of the plaque by St-EEF increases the propensity of the peptides to reaggregate after the EEF subsides.

The qualitative visual inspection of longer interpeptide separation distances during the application of the Os-EEF vs St-EEF is also corroborated by the radial distribution function (RDF) plots (see Figure S21). The plots reveal that the decomposed state of the plaque induced by St-EEF has a higher radial distribution function, [*g*(*r*)], value than its Os-EEF counterpart, indicating a lesser propensity for peptide−peptide separation by St-EEF.

### 3.4.1 Origins of St-EEF vs Os-EEF Effects

These distinct separation modes of the two EEF types reflect their contrasting modes of operation. The Os-EEF phases undergo rapid alternations as well as instantaneous changes in the magnitude of the applied field with the progress of the simulation, as governed by the eq 1.^62,63,69^ Here, *E*(*t*) is the magnitude of the electric field at time *t*, *E*_o_ represents the maximum amplitude, which is 0.02 V/Å in our case, ω is the angular frequency defined as 2π*f*, where *f* is the applied frequency, and ϕ is the initial phase, which is zero in our simulation.
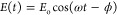
1

As a result, a given aggregation mode undergoes stabilization, followed by destabilization due to the altered phase as well as the instantaneous changes in the effective magnitude of the field. During exposure to the Os-EEF, the magnitude of the effective field strength changes at each time step, as dictated by eq 1, in addition to the change in phase. This alteration results in an instantaneous adjustment of the stabilizing interaction energy, which arises from the interaction between the magnitude of the effective electric field and the dipole moments of the peptides (see eq 2, later in Section 3.6).

Consequently, all intermolecular weak interactions, which facilitate peptide aggregation, experience continuous changes in external perturbation energies, ultimately hindering the attainment of equilibrium conditions and making the amyloid plaque very unstable. As such, once the field changes phase, it becomes easier to disintegrate the peptides. These two combined effects give rise to the observed efficient decomposition as well as the larger interpeptide separation (see Figures 1b and 2a).

By contrast, St-EEF maintains a constant phase as well as a consistent magnitude throughout the exposure and reconstructs the 10-peptide ensemble to an **OPP** structure, which is stabilized by the St-EEF. Using the St-EEF strength and the **OPP** dipole moment, the stabilization of this structure exceeds 108 kcal/mol.^41^ As such, the **OPP** enjoys a certain degree of decomposition resistance despite the dipole−dipole repulsion between the members of each parallel peptide pair (see Figure 7). Furthermore, since the **OPP** have large individual dipole moments, 125 D each,^41^ the St-EEF can rotate the individual peptides and disrupts the pairing. But the St-EEF cannot toss the peptides to long distances apart. Alternatively, the separation of **OPP** under the St-EEF can also be correlated with the molecular drift resulting from electrophoretic mobility. This is further discussed in Section 3.5, which considers the elementary HT pair.

### 3.4.2 Why Does the Impact of Os-EEF Depend on the Oscillation Frequency?

At this point, one may ask if the change in magnitude and phase is the ultimate cause for the superiority of the Os-EEF; why, then, do the plaques behave differently at higher frequencies such as THz? This can be understood by considering that the time period for completing one oscillation at the 1 THz frequency is 1 ps (10^−12^ s). *Due to this extremely short time period, the electric field oscillates too quickly for the large peptide molecules, which do not respond effectively*. As such, in practice, when the field’s frequency reaches the THz scale, the peptides’ motions are too slow to respond, and the plaque experiences the Os-EEF as though this was effectively a St-EEF.

### 3.5 Interaction of EEFs with the Elementary Unit of the Plaque: The HT Dimer

**Figure 8:**
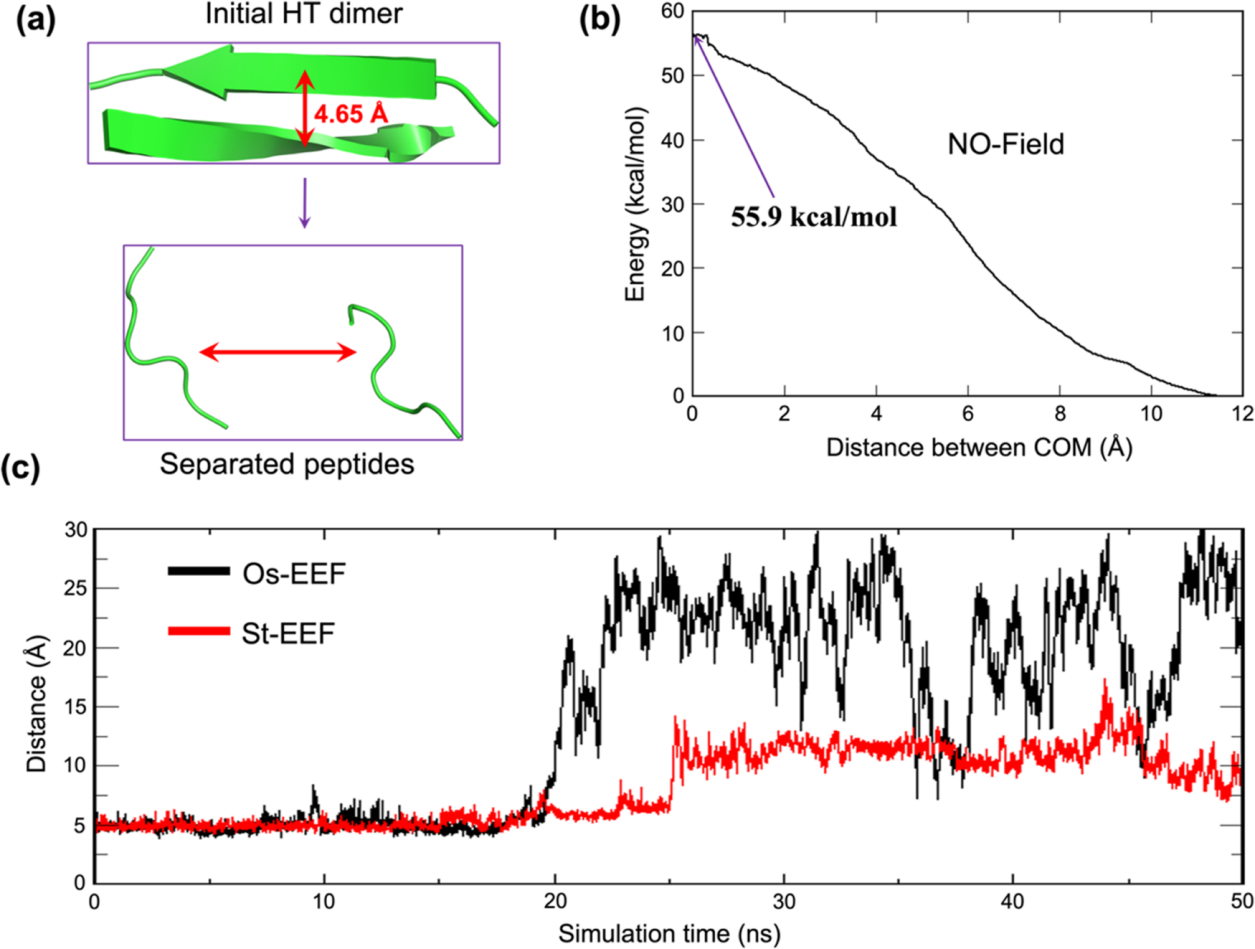
(a) HT dimer and its transformation into two separated peptides in the absence of EEF using umbrella sampling. (b) Potential of mean force (PMF) energy change for the separation of the HT unit, plotted against the distance between the centers of mass (COM), as the separation coordinate. The distance between the COM in the intact HT dimer is defined as zero, and the energy landscape is calculated until the interaction energy reaches zero. (c) Evolution of the interpeptide distance with the progress of the simulation under EEFs, starting from the initial distance of 4.65 Å between the backbone carbon atoms of two phenylalanine residues of the HT dimer in part (a). It is seen that the Os-EEF (in black) separates the dimer to an exceedingly longer distance than the St-EEF (in red).

To derive a quantitative understanding of the actions of the two EEF types, we focused on the behavior of the elementary constituent of the plaque, the head-to-tail (HT) dimer, in Figure 8a.

### 3.5.1 Binding Energy of the HT Dimer

The resulting PMF plot, in Figure 8a,b, illustrates that the HT dimer is bonded by ca. 56 kcal/mol, and this amount will have to be invested to disintegrate the HT dimer into individually separated peptides. MD simulations in the absence of the EEF revealed that the HT dimer remains stable and decomposition is not a feasible process. This observation necessarily means that the 10-peptide plaque (five HT dimers) is highly stable in the absence of an EEF.

### 3.5.2 Impacts of the Os-EEF vs St-EEF on the HT Dimer Decomposition

As shown in Figure 8c, the St-EEF separates the HT dimer to a distance of approximately 12−13 Å. In comparison, Os-EEF nearly doubles the separation between the peptides, with an average distance of ∼25 Å. This comparison emphasizes the superiority of Os-EEF over St-EEF: the Os-EEF induces a very large separation, and thereby, it diminishes the reaggregation probability after the field has subsided.

Let us focus on the quantitative comparison of the Os-EEF vs St-EEF effects using two sets of simulations for the HT dimer, which is the elementary constituent of the plaque. As expected, the decomposition of the HT dimer occurs in both EEF types but at different rates. Using our Python code (page S22 in the SI), we quantified the decomposition patterns. We found that the decomposition rate in the presence of Os-EEF is **6.33 times faster** than the rate of separation by St-EEF. This quantified relative separation -rate, in addition to the separation distances discussed above, demonstrates the significant impact of the phase and effective magnitude change in Os-EEF. This further ascertains the superiority of Os-EEF over St-EEF and confirms thereby the occurrence of ‘**plaque explosion**’ in the presence of Os-EEF. This is in stark contrast to the behavior observed with St-EEF, which decomposes the plaque in a sluggish manner, leading to smaller interpeptide separation. At the same time, let us emphasize that the impact of the Os-EEF is best in the rather low-frequency range (≤GHz), and it requires short pulses, which prevent reaggregation.

### 3.6 Decomposition of the Aβ−42 Trimer by the Two EEF Types

**Figure 9:**
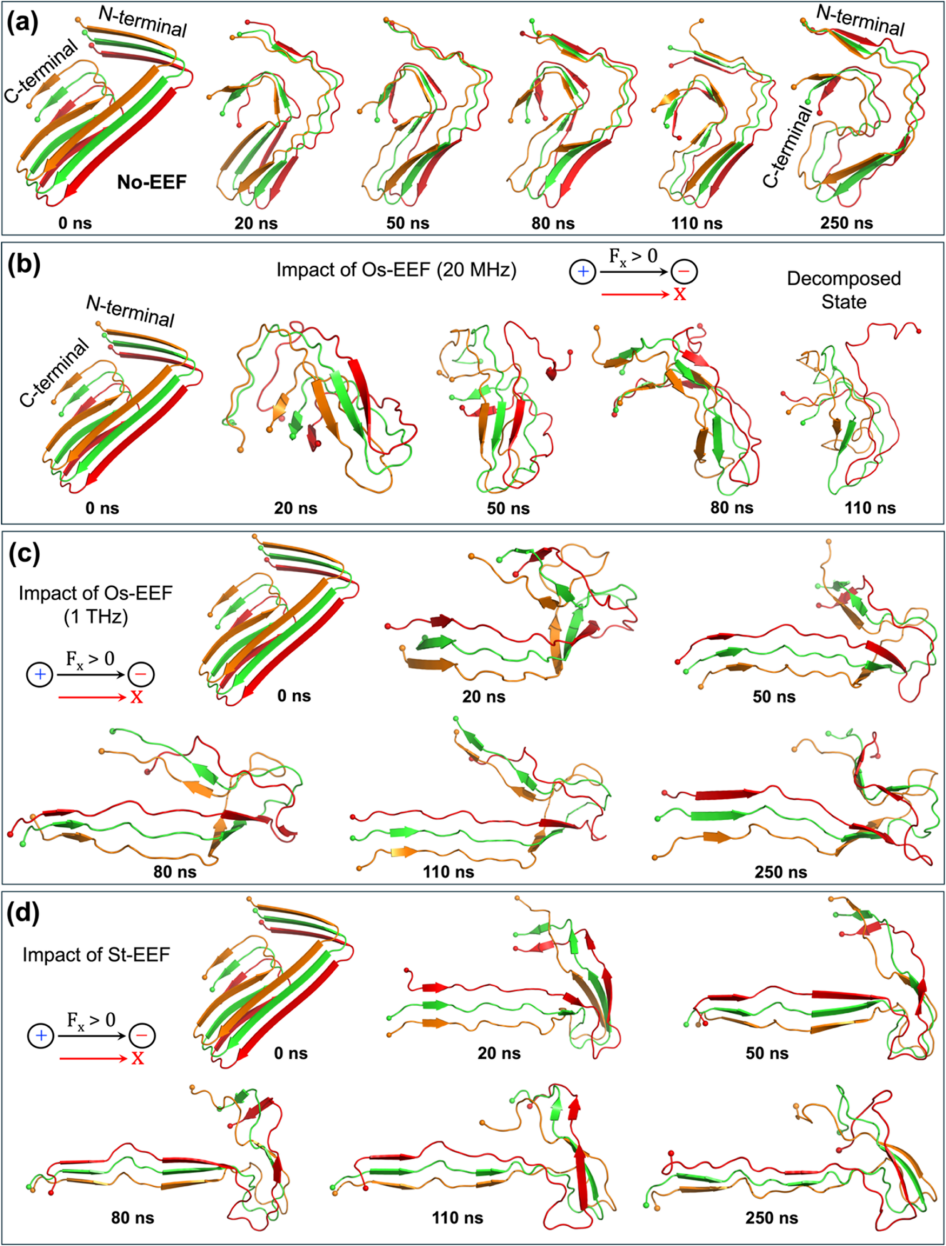
Conformational changes of the Aβ-42 trimer observed at different time scales upon application of the following simulations: (a) No-EEF and (b) Os-EEF (0.02 V/Å) at 20 MHz, with the decomposed state at 110 ns; (c) Os-EEF (0.02 V/Å) at 1 THz; and (d) St-EEF (0.02 V/Å). In all cases, terminals of the peptides are marked with a “ball” representation for clear distinction.

This section describes how an Os-EEF of moderate-to-low frequencies (≤GHz) effectively decomposes an Aβ-42 trimer, compared to the sluggish impacts of St-EEF and a high-frequency Os-EEF (≥THz). Before applying the EEF, we first ensured the intrinsic stability of the Aβ-42 trimer (see Figure 9a). The number of hydrogen bonds and total β−sheet content, which remain constant throughout the simulation, indicates that the Aβ-42 trimer remains stable in the absence of EEF (see also Figure 11 later).

The Aβ−42 trimer in Figure 9a was subsequently subjected to an EEF of 0.02 V/Å under three different conditions shown in Figures 9b–d: Os-EEF at 20 MHz, an Os-EEF at 1 THz, and a St-EEF. As shown in Figure 9b, upon the application of an Os-EEF at 20 MHz, the Aβ−42 trimer began to lose its initial conformation by 20 ns. Subsequently, without much exploration of the potential energy surface, and similar to the “plaque explosion” observed in the 10-peptide plaque, the Aβ-42 trimer quickly loses its structural integrity (at ∼110 ns), marking an “explosive” decomposition to a **disordered state**.

In contrast, using 1 THz (see Figure 9c) or St-EEF (see Figure 9d), the Aβ−42 plaque does not decompose, aside from minor conformational changes. Thus, as shown in Figure 9c,d, rather than undergoing a complete deformation, the Aβ−42 trimer units opened their trademark S-shaped fold (see 0 ns vs 110 ns) with an attempt to align along the direction of the applied EEF. Furthermore, Figures S22 and S23 (in the SI) show representative interpeptide distances and their evolution over the course of the simulation under all three conditions (Os-EEF at 20 MHz, Os-EEF at 1 THz, and St-EEF), illustrating how the tripeptides separate as the simulation progresses.

The inability of a THz frequency to decompose either the short peptide plaque or the Aβ−42 trimer is similar to the observation described by Chen et al.,^36^ who demonstrated that a specific THz frequency (42.55 THz) mildly stabilizes tetrameric protofibrils at an extremely low EEF strength (0.5 V/m). By comparison, our simulation offers a broad perspective on the effects of frequency by considering a relatively higher EEF (0.02 V/Å). Despite this higher EEF, we observe that the THz frequency still fails to decompose the short peptides’ plaque or deform the trimer, similar to the effects seen with moderate- to low-frequency fields.

**Figure 10:**
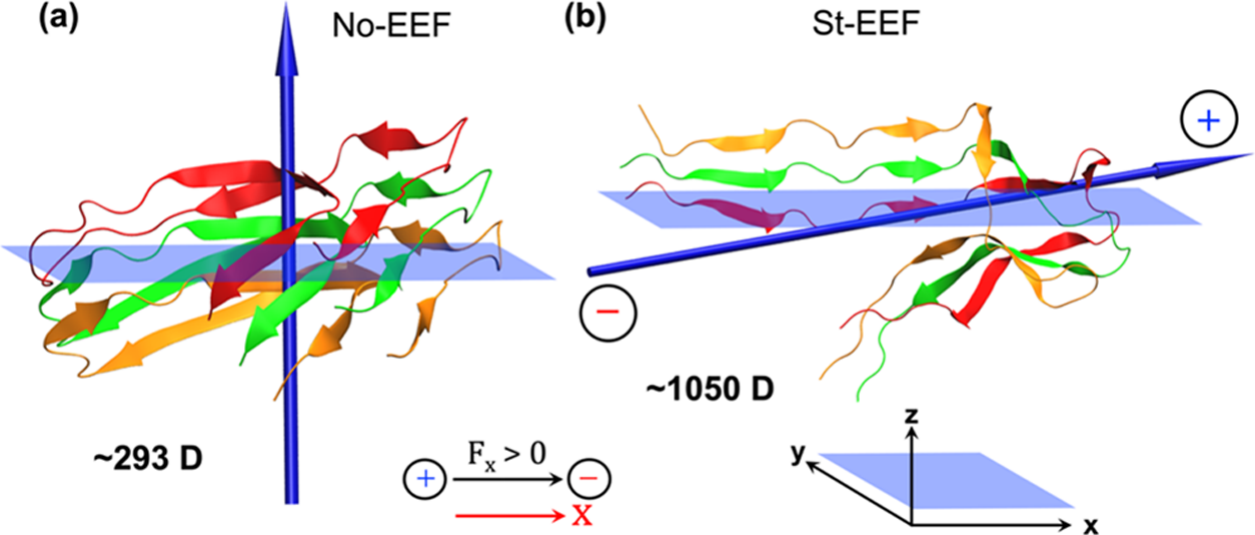
Dipole moment direction of the Aβ-42 trimer, captured after 110 ns of simulation, is represented as follows: (a) in the No-EEF condition and (b) during the St-EEF condition. The transparent blue rectangle represents the “*xy*” plane, where the “*x*“ axis denotes the direction of the EEF. This is provided to better correlate the direction of the EEF with the dipole moment of the Aβ-42 trimer, which is indicated by the blue cylindrical arrow.

As shown in Figure 10, these conformational changes are primarily governed by the interaction between the resultant dipole moment (μ) of the Aβ-42 trimer and the applied EEF (F_*x*_). Thus, as shown in Figure 10a, the dipole moment of the Aβ-42 trimer is approximately 293 D in the absence of EEF. When St-EEF or Os-EEF at 1 THz is applied, the peptides reorient and open the trademark S-shaped configuration (see Figure 9c,d) to maximize their dipole moment (on average 1050 D; cf. Figure 10b), aligning it almost in parallel to both the applied EEF and the peptide plane. Thus, the St-EEF and Os-EEF (at the THz frequency) change the conformation of the Aβ-42 trimer and enhance its interaction with the respective EEFs.

As such, the new parallel conformation of the Aβ-42 trimer (see Figure 10b) interacts favorably with the EEFs, achieving a stabilization energy of 100.8 kcal/mol, as shown in eq 2.
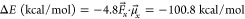
2

Thus, this newly formed conformation becomes stable under St-EEF, which prevents the trimer from disintegrating into a disordered state. On the other hand, due to the phase and magnitude changes (see eq 1) of the Os-EEF (≤GHz), the stabilization energy (Δ*E*) varies instantaneously, as described by eq 2, and additionally, once the Os-EEF changes its phase, it causes the peptides to realign in the opposite direction. Consequently, these combined effects lead to severe instability in the Aβ−42 trimer under an Os-EEF (≤GHz), *resulting in a rapid deformation of the trimer into a highly disordered state*, as observed at ∼110 ns in Figure 9b.

**Figure 11:**
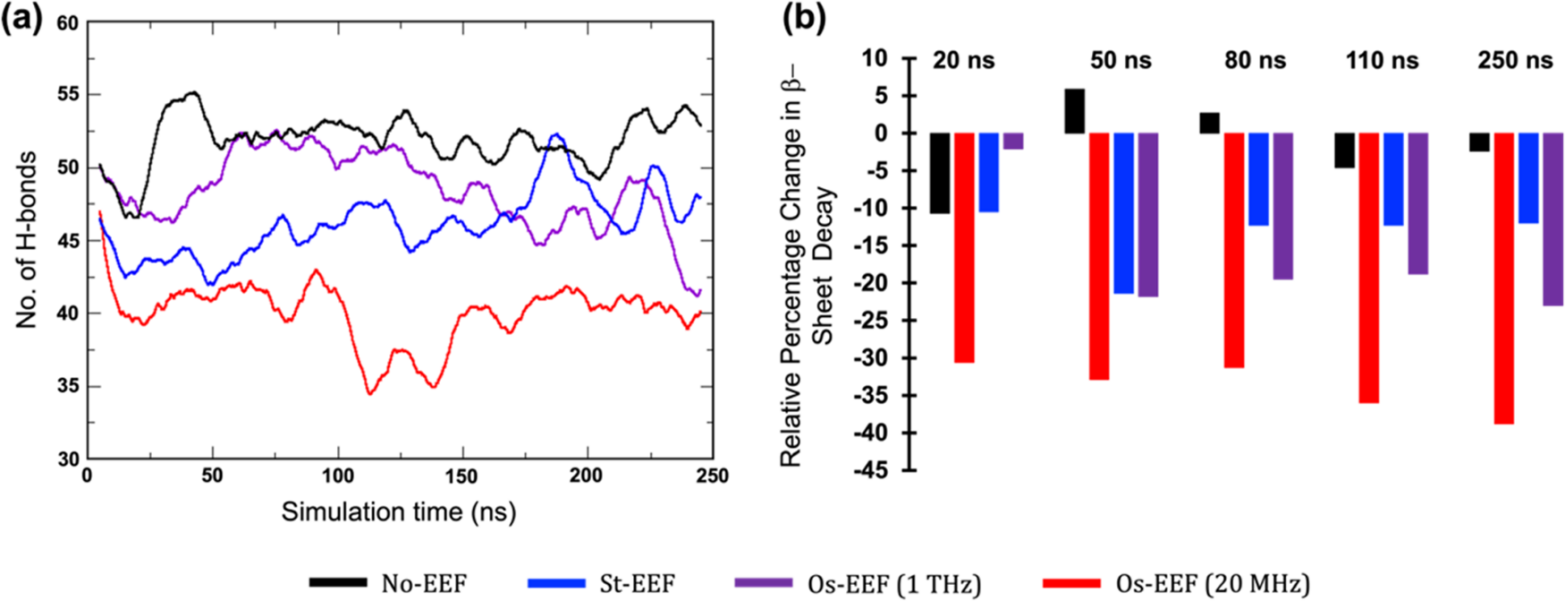
(a) Evolution of hydrogen bonding with the progress of the simulation. All plots have been transformed using “running averages” over 1000 points (total points: 25000) in XMGRACE software to provide a clearer correlation among them. (b) Bar diagram showing the percentage changes in β−sheet decay relative to the initial conformation, supporting the visual observations in Figure 9 across each time scale. All values at different time scales were determined using the DSSP algorithm incorporated in AMBER22 software. The raw hydrogen bonding and 2D-DSSP plots are included in the SI (see Figures S24 and S25).

Furthermore, the decomposition pattern of the Aβ-42 trimer under different conditions can be realized from the evolution of hydrogen bonding and the relative percentage change in the β-sheet content, which is the basic secondary structure that maintains the integrity of the Aβ-42 trimer. As shown in Figure 11a, the number of hydrogen bonds is marginally lower in the case of Os-EEF at 20 MHz (red) compared to St-EEF (blue), Os-EEF at 1 THz (purple), and No-EEF (black) conditions, providing evidence of a decomposition event. The fewer hydrogen bonds in the cases of St-EEF and Os-EEF at 1 THz compared to the No-EEF simulation also indicate the occurrence of slight conformational changes in the Aβ-42 trimer upon application of EEF.

Figure 11b illustrates the relative percentage change in the β−sheet decay. This measure supports the visual data presented in Figure 9 across different time scales and assesses quantitatively the impact of the EEF types. The red bar, indicating the 20 MHz frequency, shows the highest β-sheet decay at all time scales. In contrast, the purple and blue bars, representing 1 THz and St-EEF, exhibit reduced β-sheet decay, clearly indicating a lower efficiency in disrupting the Aβ−42 trimer. A detailed analysis of secondary structure evolution over time can be seen in the 2D-DSSP plot in the SI (see Figure S25). Conversely, the black bar, corresponding to the No-EEF simulation, displays minimal changes in β−sheet decay, preserving the original conformation of the Aβ−42 trimer.

Hence, the above descriptions clearly demonstrate that an Os-EEF with moderate-to-low frequencies (≤GHz) can decompose the Aβ-42 trimer “explosively,” similar to what was observed in the 10-short-peptide plaques. On the other hand, the impact of Os-EEF at the ≥ THz frequency range resembles the impacts of St-EEF.

### 3.7 Experimental Relevance of the EEF Strengths

The preceding sections discussed various mechanistic aspects associated with the aggregates of Aβ peptides in the presence of EEFs with a strength of 0.02 V/Å. The present section describes the impact of weaker fields on the decomposition of the Aβ aggregates. As noted in Section 2.3, we found that decreasing the EEF strength still leads to plaque decomposition, albeit at longer simulation time scales.

**Figure 12:**
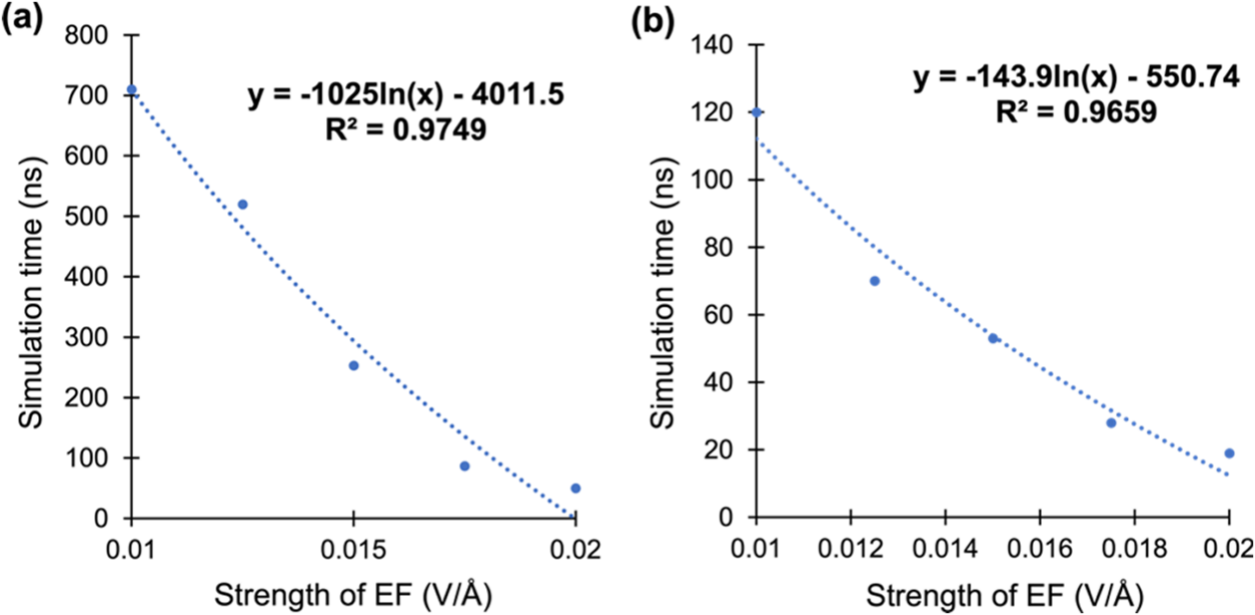
Correlation plots (a) for the short peptide plaque and (b) for the HT dimer, showing the relationship between the time required for their decomposition at EEF strengths ranging from 0.02 to 0.01 V/Å. The dotted curve represents the correlation of the various points and the fitted equation of the curves (in the top-right corners).

As shown in Figure 12a, fitting the data points for the time scales of the simulations vs the strength of the applied EEF provides a mathematical expression, *which can predict the decomposition time scale for plaques at lower EEF strengths*. According to the equation displayed in Figure 12a, we found that the decomposition time scale for 10-peptide plaques at a field strength of 3 × 10^−6^ V/Å, as used by Saikia et al.,^43^ is approximately 9.1 μs.

To show the unity of the predictions, we tested the mathematical models (in Figure 12a) using the HT dimer in Figure 12b. Doing so, we verified that an EEF strength of 3 × 10^−6^ V/Å decomposes the HT dimer on a time scale approximately 60 times longer (∼1203 ns) than the time scale for the much stronger EEF, the 0.02 V/Å (20 ns). This time scale is also closer to the predicted value (∼1279 ns) using the equation in Figure 12b. As such, our modeling bridges the gap between computationally applicable and experimentally viable EEF strengths (see detailed systematic results in the SI; Section S.4 and Figures S5−S6).

### 3.8 Similarities Between the EEF Approach and TTFields Therapy

One of the main goals of the current and previous studies^41^ is to propose an EEF-based potential therapeutic model for Alzheimer’s disease, similar to TTFields therapy.^26−30^ Therefore, it is important to summarize how our computational findings align with the principles of TTFields therapy, as outlined below.

(a) Our simulations suggest that Os-EEF is more effective than St-EEF in decomposing β−amyloid plaques. This finding mirrors the fundamental strategy of TTFields therapy, which uses alternating electric fields in its tumor treatments.
(b) Similar to the noninvasive nature of TTFields therapy, our proposed use of Os-EEF also emphasizes a noninvasive approach for disrupting β−amyloid plaques, highlighting its potential translational relevance.
(c) Both TTFields therapy and our proposed approach share a mechanistic foundation involving the modulation of dipole moments. In TTFields, the electric fields interact with microtubules,^70−72^ whereas in our present work, the electric field targets β-amyloid aggregates, underscoring a shared principle in the therapeutic actions.
(d) TTFields therapy is primarily used to treat solid brain tumors. Similarly, our study focuses on β−amyloid plaques, which also form in the brain, thereby broadening the scope of electric field-based interventions in brain pathologies.

## 4 Conclusions

The primary objective of this study was to investigate the root cause of the superiority of the Os-EEF over the St-EEF in decomposing senile plaques. Our findings revealed that Os-EEFs lead to “**plaque explosion**,” which spreads apart the individual peptides to long distances. By contrast, the application of St-EEFs results in a sluggish dispersion typified by relatively short interpeptide distances and an apparent reversibility at the same time scale as decomposition.

Furthermore, our study explored comprehensively the root cause of “plaque explosion” caused by the Os-EEF. We show that the explosion originates in the inherent phase and effective changes of field strength during the cycles of the Os-EEF and its impact on the interactions among the peptides in the β−amyloid plaque. By contrast, the lack of phase and magnitude change in the presence of St-EEF is the root cause for the absence of such an explosive event of the plaque and for the reversibility of the decomposition event. Our simulation also shows that the Os-EEF at very high frequencies (≥THz) behaves similarly to the St-EEF as the movements of the protein parts are too slow to follow the field’s oscillation frequency. This observation further supports the use of relatively low-frequency fields that are experimentally feasible and medically safer. Moreover, replicating these observations with a full-length Aβ−42 trimer provides additional insight into how the Os-EEF and St-EEF may act in decomposing entire full-length Aβ−42 senile plaques.

The present work also explores the impact of prolonging the application of an Os-EEF after the explosive decomposition of the plaque. Our findings indicate that under the influence of the field, the individually separated peptides initiate the formation of parallel pairs (**PP**). However, the process of **PP** formation is disrupted once again under the continued influence of the Os-EEF. We further believe that all of the above findings can be experimentally validated through in situ studies, such as ThT and Congo Red fluorescence measurements, FRET, fluorescence correlation spectroscopy, etc.

In conclusion, therefore, our study supports the preference for use of the Os-EEF in treating senile plaque-related diseases. **The optimal application of Os-EEFs is recommended in short pulses**, which not only decompose the plaque but also avoid the formation of **PP** (parallel pairs). As such, our study highlights a potential therapeutic concept aligned with TTFields, which is the Os-EEF-based-therapy currently used for solid tumors.^26−30^

## Supporting Information Available

A comprehensive description of various MD simulation parameters, a detailed discussion of umbrella sampling procedures, the root-mean square deviation (RMSD) plot for the peptide backbone atoms, evolution of hydrogen bonding with progress of the simulation, the decay of secondary structure of peptides over time, the radial distribution function (RDF) plot, evolution of interpeptide distances, evolution of radius of gyration, percentage decay of β−sheet with time, mathematical modeling, and various other MD snapshots (PDF)

## Abbreviations

EEF: external electric field
Os-EEF: oscillating external electric field
St-EEF: static external electric field
HT: head-to-tail
PP: parallel pairs
OPP: organized parallel pairs
RPP: random parallel pairs
AP: antiparallel
