# Supplementary material for: Origins of the Superiority of Oscillating Electric Fields for Disrupting Senile Plaques: Insights from the 7-Residue Fragment and the Full-length Aβ-42 Peptide
Source: J Am Chem Soc. 2025 Jan 8;147(3):2626–41. doi: 10.1021/jacs.4c14791 (PMC11760182; doi:10.1021/jacs.4c14791)
Supplement: Supplementary file 1 — ja4c14791_si_001.pdf [file ja4c14791_si_001.pdf]

Supporting Information

Origins of the Superiority of Oscillating Electric Fields for Disrupting Senile Plaques: Insights from the 7-Residue Fragment and Full-length Aβ−42 Peptide

Surajit Kalita*, David Danovich and Sason Shaik*

Institute of Chemistry, The Hebrew University of Jerusalem, Edmond J. Safra Campus, Givat Ram, Jerusalem 9190401, Israel
Email: sason@yfaat.ch.huji.ac.il; sason.shaik@gmail.com

Table of Contents:

S.1. System Preparation of Aβ−42 Trimer **S4**

S.2. Detailed Description of Simulation Parameters **S4**

S.3. Reduction in the Strength of Electric Field Below 0.02 V/Å: Os-EEF vs St-EEF **S5–S7**

S.4. A Mathematical Model for Predicting Explosion Time at Lower Field Strengths **S7–S11**

S.5. Analysis of the Heating Effects **S11**

S.6. Procedures for the Umbrella Sampling Simulations **S12**

S.7. Alternative Descriptions of the Rapid Decomposition of Plaques **S13–S14**

S.8. Periodic Buildup and Destruction of Parallel Pairs **S14–S15**

Figure S1: The starting conformation of the Aβ-42 fibril (PDB ID 5OQV) and the trimer derived from it are shown. The growth axis of the fibril is perpendicular to the plane of the paper. **S4**

Figure S2: (a) Snapshot showing the occurrence of plaque explosion in the presence of Os-EEF of strength 0.0175 V/Å with frequency 0.1 GHz. (b) Snapshot showing the conformational alterations of amyloid plaque under St-EEF of strength 0.0175 V/Å. Both snapshots were taken after 87 ns of simulation time. The double-headed arrows represent the interpeptide distances in Å. Panels (c) and (d) represent the evolution of the radius of gyration (R_g) with simulation time in the presence of Os-EEF and St-EEF, respectively. **S5**

Figure S3: (a) Snapshot showing the occurrence of plaque explosion in the presence of Os-EEF of strength 0.015 V/Å with frequency 0.1 GHz. (b) Snapshot showing the conformational alterations of amyloid plaque under St-EEF of strength 0.015 V/Å. Both snapshots were taken after 253 ns of simulation time. The double-headed arrows represent the interpeptide distances in Å. Panels (c) and (d) represent the evolution of the R_g with simulation time in the presence of Os-EEF and St-EEF, respectively. **S6**

Figure S4: Snapshots showing the plaque explosion in the presence of Os-EEF at a frequency of 0.1 GHz are presented with strengths of (a) 0.0125 V/Å and (b) 0.01 V/Å. The corresponding evolution of R_g is shown in panels (c) and (d), respectively **S7**

Figure S5: (a) Graphical representation of an HT dimer before and after its separation. The interpeptide distance plots show the evolution of the HT dimer at different field strengths: (b) 0.0175 V/Å, (c) 0.015 V/Å, (d) 0.0125 V/Å, and (e) 0.01 V/Å. For calculating the interpeptide

distance, we selected the backbone carbon atoms of two phenylalanine residues, as shown in Figure a. **S8**

Figure S6: (a-c) Different conformations of the HT dimer obtained at various timescales during simulation. (d) The evolution of the interpeptide distance of the HT dimer at 3×10^{-6} V/Å. Similar to other simulations, the interpeptide distance was calculated by selecting the backbone carbon atoms of two phenylalanine residues, as shown in Figure 5a. Panel (e) shows the evolution of the interpeptide distance between the Glu-Glu residues. **S9**

Figure S7: Evolution of temperature under different conditions, starting from 300 K. **S11**

Figure S8: (a) Uniform evolution of distance between the COM of both peptides with progress of the simulation. (b) Depiction of significant overlap of windows simulated for umbrella sampling. **S12**

Figure S9: (a) The RMSD plot highlights the acquired instability in the plaque during the initial 50 ns timescale for the Os-EEF with frequency 0.1 GHz and strength 0.02 V/Å. (b) Evolution of the number of hydrogen bonds for the first 50 ns of simulation time. (c) Evolution of the interpeptide distance (in Å) with the progress of the simulation for a replica simulation in the identical condition focusing on the backbone carbon atoms, as shown in Figure 1a. **S13**

Figure S10: Decay of the secondary structure with progress of the simulation at 0.1 GHz frequency with EEF strength 0.02 V/Å. Each 9-residue index refers to a single peptide chain. **S14**

Figure S11: (a) Formation and destruction of a parallel-pair (PP) structure, which is formed due to a prolonged exposure to Os-EEF (0.01 GHz with 0.02 V/Å) after the plaque explosion. The initial coordinates at 0 ns were taken from Figure 6b. All double-headed arrows indicate interpeptide backbone-backbone distances in Å. (b) Evolution of interpeptide distance within the PP during the simulation. The separation is calculated using the backbone carbon atoms of a phenylalanine pair. **S14**

Figure S12: RMSD plot for the simulation of parallel pair under Os-EEF (0.01 GHz with 0.02 V/Å), showing the sudden instability after around 230 ns. **S15**

Figure S13: Evolution of the number of hydrogen bonds with progress of the simulation in the presence of Os-EEF at 0.02 V/Å and a frequency of 20 MHz. **S15**

Figure S14: The evolution of (a) the radius of gyration and (b) the number of hydrogen bonds in 10-peptide plaques as the simulation progresses, under the influence of an Os-EEF of 0.02 V/Å with a frequency of 20 MHz. (c) Evolution of the interpeptide distance (in Å) with the progress of the simulation in the identical condition focusing on the backbone carbon atoms, as shown in Figure 1a. **S16**

Figure S15: (a) The decomposition of a 10-peptide plaque at 50 ns, due to exposure to an Os-EEF (along the x-axis) of 0.02 V/Å with a frequency of 500 kHz. The double-headed arrows indicate several representative interpeptide distances (in Å) among the dispersed peptides. The black arrow in the top right corner marks the direction of the applied Os-EEF. (b) The corresponding RMSD of backbone atoms over the course of the simulation. **S16**

Figure S16: Evolution of the number of hydrogen bonds with progress of the simulation in the presence of Os-EEF at 0.02 V/Å and a frequency of 1 THz. **S17**

Figure S17: The evolution of (a) the radius of gyration and (b) the number of hydrogen bonds in 10-peptide plaques as the simulation progresses, under the influence of an Os-EEF of 0.02 V/Å with a frequency of 1 THz. **S17**

Figure S18: The evolution of (a) the radius of gyration and (b) the number of hydrogen bonds in 10-peptide plaques as the simulation progresses, under the influence of an Os-EEF of 0.02 V/Å with a frequency of 10 THz. **S18**

Figure S19: Percentage of β-sheets decay in the presence of Os-EEF with frequency 0.1 GHz at a strength of 0.02 V/Å. Each data point represents the change in β-sheet percentage relative to the initial value observed in the amyloid plaque at 0 ns. **S18**

Figure S20: The evolution of the radius of gyration of 10-peptide plaques as the simulation progresses, in the presence of an St-EEF with a strength of 0.02 V/Å. **S19**

Figure S21: Peptide-peptide radial distribution function (RDF) plot of the initial plaque and its decomposition by St-EEF as well as Os-EEF. Calculations are done using the AMBER inbuilt algorithm, focusing specifically on the backbone carbon atoms and utilizing data from the entire trajectory Note that the first hump primarily reflects the intrapeptide separation, originating from the interactions between backbone carbon atoms within the same peptide. The second hump corresponds to the interpeptide separation, indicating the distances between peptides. Notably, the initial plaque (black curve) exhibits the highest RDF, followed by the St-EEF (red curve), and the Os-EEF simulation (green curve) displays the lowest RDF. As the Os-EEF RDF is the lowest, it suggests greater peptide-peptide separation compared to St-EEF. **S19**

Figure S22: The conformations of the Aβ-42 trimer are shown as follows: (a) at 0 ns in the absence of EEF, (b) and (c) at 110 ns with Os-EEF applied at 1 THz and 20 MHz, respectively, and (d) at 110 ns with St-EEF. All conditions have an identical EEF strength of 0.02 V/Å. The double-headed arrows indicate the representative interpeptide distances in Å between the peptides. **S20**

Figure S23: The evolution of interpeptide distances observed under different conditions is illustrated as follows: (a) represents the terminal-terminal distances, indicated by point (A) in Figure S22a, while (b) shows the non-terminal interpeptide distances, marked by point (B) in Figure S22a. **S20**

Figure S24: Evolution of the number of hydrogen bonds throughout the simulation under various conditions: (a) No-EEF, (b) Os-EEF with a frequency of 1 THz, (c) St-EEF, and (d) Os-EEF with a frequency of 20 MHz. In all cases, the EEF strength is 0.02 V/Å. **S21**

Figure S25: Decay of the secondary structure throughout the simulation under various conditions: (a) No-EEF, (b) Os-EEF with a frequency of 1 THz, (c) St-EEF, and (d) Os-EEF with a frequency of 20 MHz. The plots were generated from the trajectory using the AMBER-built CPPTRAJ program with the DSSP algorithm for secondary structure analysis. **S22**

Python Code for Velocity Calculation: **S22–S24**

References **S24**

S.1. System Preparation of Aβ–42 Trimer

The Aβ-42 trimer was derived from the fibril structure (see Figure S1), which was experimentally determined using cryo-electron microscopy. As shown in Figure S1, three adjacent Aβ-42 peptides were selected to generate the Aβ-42 trimer.

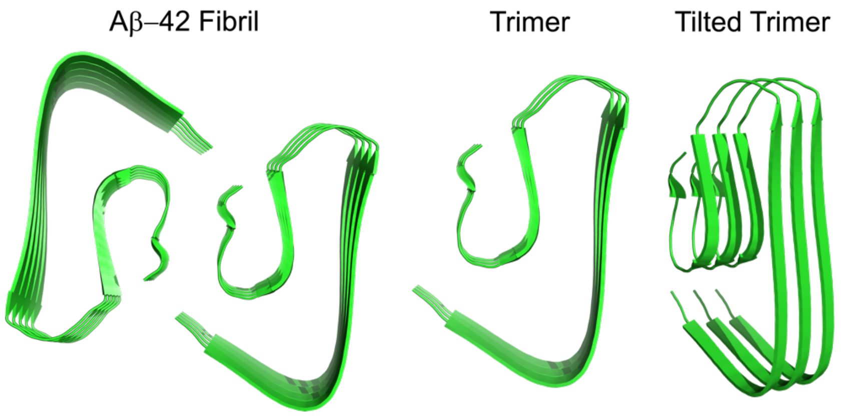

Figure S1: The starting conformation of the Aβ-42 fibril (PDB ID 5OQV) and the trimer derived from it are shown. The growth axis of the fibril is perpendicular to the plane of the paper.

The missing hydrogen atoms of the Aβ-42 trimer were added using the LEAP module of AMBER22 with the ff14SB force field. Thereafter, the entire system was placed in an octahedral box of TIP3P water, extending up to 10 Å from the peptide surface. The overall charge of the system was neutralized with the appropriate number of Na^+ ions.

S.2. Detailed Description of Simulation Parameters

After system setup, we subjected our peptide-systems to a two-step minimization process. Initially, solvent minimization was conducted, followed by a subsequent full system minimization with no restraints. This minimization involved 5000 steps of the steepest descent method, followed by 5000 steps of the conjugate gradient algorithm. Gradual heating of the system from 0 to 300 K were carried out over 50 ps, employing the NVT ensemble. Subsequently, the system underwent a 1 ns simulation under the NPT ensemble at a target temperature of 300 K and a pressure of 1.0 atm. The Langevin thermostat[1] was used with a collision frequency of 2 ps, and the Berendsen barostat[2] was applied with a pressure relaxation time of 1 ps. We further equilibrated our systems for 3 ns by applying the same protocol. After completion of the equilibration of all systems, we performed the production MD runs employing the NVT ensemble. Monte Carlo barostat[3] was used for the production MD simulation. The replica simulations were conducted to ensure the consistency of the obtained results. The rapid movement of hydrogen bond was constrained using the SHAKE[4] algorithm.

While treating the long-range electrostatic, we employed the Particle Mesh Ewald (PME) algorithm[5] and a cutoff of 12 Å was chosen for van der Waals forces. During all production MD simulations, we applied the Periodic Boundary Condition (PBC) in a systematic way to reduce the edge effects of the simulation cell. Additionally, for the simulation in the presence of EEF, we adjusted the intensity of the applied field using the built-in keyword 'efn=1' in AMBER22.

S.3. Reduction in the Strength of Electric Field Below 0.02 V/Å: Os-EEF vs St-EEF

We conducted a comparative analysis of St-EEF vs Os-EEF to understand the behavior of amyloid disintegration when exposed to an EEF of strength lower than 0.02 V/Å. Accordingly, we performed two sets of simulations under St-EEF and Os-EEF conditions at strengths of 0.0175 V/Å, 0.0150 V/Å, with a frequency of 0.1 GHz for all Os-EEF simulations. We further extended the Os-EFF simulations with two additional sets, at 0.0125 V/Å and 0.01 V/Å, to understand the trend in decomposition with further reduction in EF strengths. Below, we systematically discuss the results obtained from all simulations.

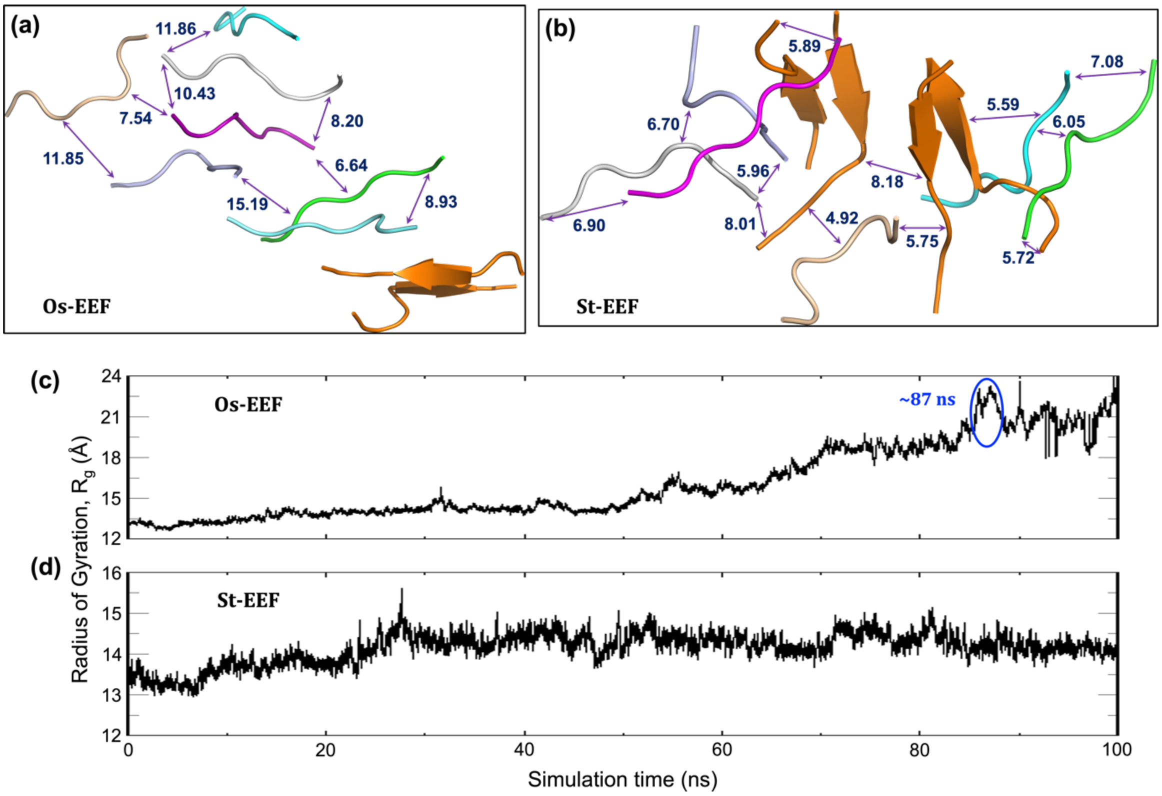

Figure S2: (a) Snapshot showing the occurrence of plaque explosion in the presence of Os-EEF of strength 0.0175 V/Å with frequency 0.1 GHz. (b) Snapshot showing the conformational alterations of amyloid plaque under St-EEF of strength 0.0175 V/Å. Both snapshots were taken after 87 ns of simulation time. The double-headed arrows represent the interpeptide distances in Å. Panels (c) and (d) represent the evolution of the radius of gyration (R_g) with simulation time in the presence of Os-EEF and St-EEF, respectively.

In the presence of Os-EEF = 0.0175 V/Å, we observe that plaque explosion occurs at a slightly longer timescale of **~87 ns** (vs. 50 ns at 0.02 V/Å), as shown in Figure S2a. We do not observe such an explosion in the presence of St-EEF at the identical timescale, as shown in Figure S2b. This visual observation is further supported by the radius of gyration (R_g) plots in Figure S2c and S2d, where a gradual increase in R_g value is seen in the case of Os-EEF, while no such increase is observed in the case of St-EEF. It is to be noted that the increasing R_g value suggest the separation of peptides or the decreased compactness in the peptide aggregates.

We then further reduced the electric field strength by an additional 0.0025 V/Å increment, and conducted simulations at a strength of 0.015 V/Å. Similarly, we observe the plaque explosion phenomenon at ~253 ns in the presence of Os-EEF, as shown in Figure S3a, which is again substantiated by the R_g plot in Figure S3c, indicating a sharp and continuous rise in the R_g value. In contrast, we also observe a sharp rise in the R_g value at around 250 ns in the presence of St-EEF (cf. Figure S3d), but for a very short period of time, after which it returns to its initial value and continues to evolve in that region. Figure S3b illustrates the St-EEF inability to induce plaque explosion or separate the peptides into longer distances.

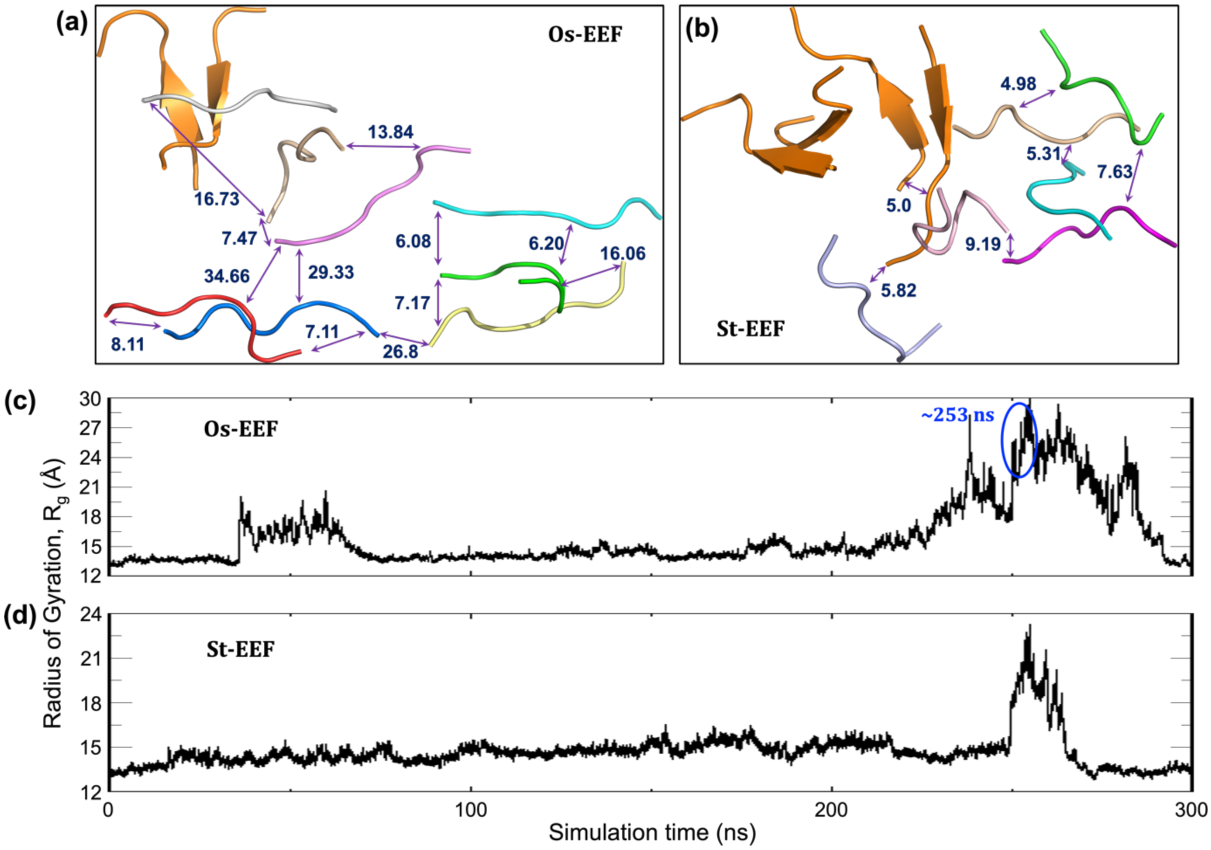

Figure S3: (a) Snapshot showing the occurrence of plaque explosion in the presence of Os-EEF of strength 0.015 V/Å with frequency 0.1 GHz. (b) Snapshot showing the conformational alterations of amyloid plaque under St-EEF of strength 0.015 V/Å. Both snapshots were taken

after 253 ns of simulation time. The double-headed arrows represent the interpeptide distances in Å. Panels (c) and (d) represent the evolution of the R_g with simulation time in the presence of Os-EEF and St-EEF, respectively.

From the simulations at 0.0175 and 0.015V/Å, it is evident that unlike St-EEF, Os-EEF can lead to the plaque explosion phenomenon even at the reduced electric field strengths, albeit occurring at a longer timescale compared to higher field strengths. Consequently, it can be anticipated that plaque explosion under Os-EEF can occur at any reduced field strength, although the simulation time will increase upon decreasing the field strength. To bolster our anticipation, we further simulated the system at 0.0125 V/Å and 0.01 V/Å in the presence of a frequency of 0.1 GHz. As expected, we observe the plaque explosion phenomenon, as shown in Figure S4a,b, and its corresponding R_g plot in Figure S4c,d.

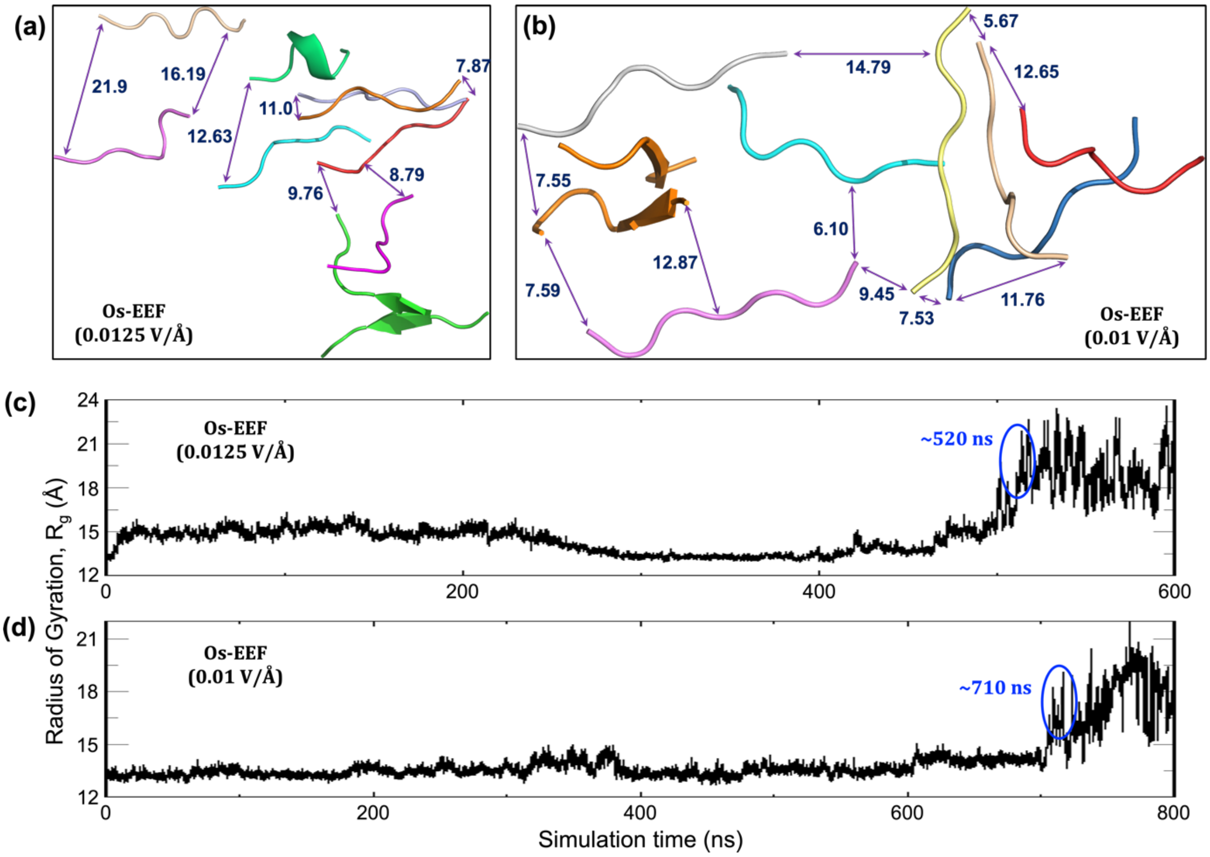

Figure S4: Snapshots showing the plaque explosion in the presence of Os-EEF at a frequency of 0.1 GHz are presented with strengths of (a) 0.0125 V/Å and (b) 0.01 V/Å. The corresponding evolution of R_g is shown in panels (c) and (d), respectively.

S.4. A Mathematical Model for Predicting Explosion Time at Lower Field Strengths

The discussion in Section S.3 demonstrates that plaque explosion occurs under Os-EEF at various reduced EF strengths. However, decomposition requires a longer simulation timescale

as the EF strength decreases, as illustrated by the correlation points shown in Figure 12a (see main text). These correlation points are fitted with a logarithmic equation **[y = −1025 ln(x) – 4011.5]**, yielding an **R^2 value of 0.9749** (see Figure 12a). Since an R^2 value close to 1 indicates an ideal fit, our data points align well with this equation. This logarithmic model describes our observation of a gradual increase in explosion time as EF strength decreases. Interestingly, we can now predict the decomposition time for any applied EEF using this equation. For example, the timescale required for the decomposition of a 10-peptide plaque at an experimentally viable field strength, **such as 3×10^{-6} V/Å, is approximately 9100 ns, or 9.1 μs**. This predicted duration is exceedingly long relative to typical simulation timescales; however, with a powerful computer, it might be possible to observe plaque decomposition at such extended timescales. Nonetheless, performing simulations over such long durations is beyond the capability of our current reasonable computational resources. As such, we believe this mathematical model bridges the gap between the EEF strength applied in our simulations and the clinically feasible EF strength.

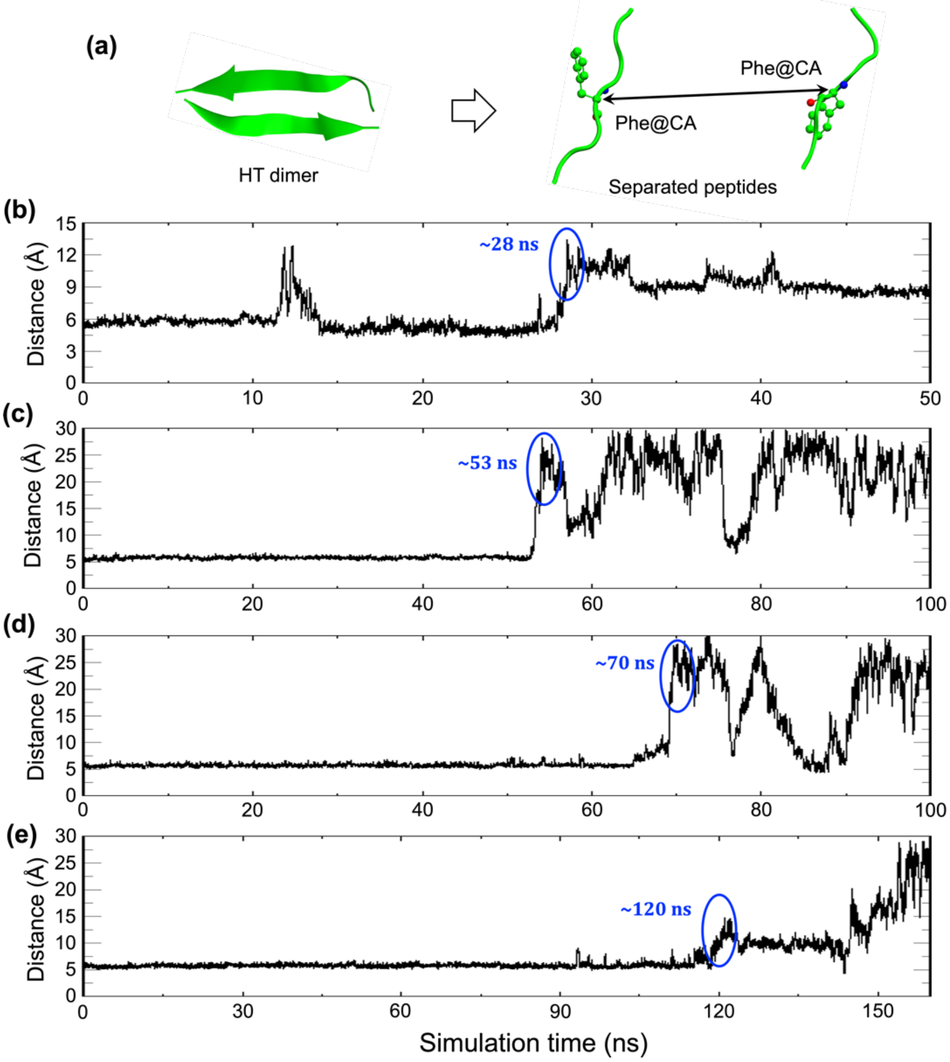

Figure S5: (a) Graphical representation of an HT dimer before and after its separation. The interpeptide distance plots show the evolution of the HT dimer at different field strengths: (b) 0.0175 V/Å, (c) 0.015 V/Å, (d) 0.0125 V/Å, and (e) 0.01 V/Å. For calculating the interpeptide distance, we selected the backbone carbon atoms of two phenylalanine residues, as shown in Figure a.

To address the predictive power of our proposed model, we tested it using an HT dimer, which is computationally less expensive compared to the modeled plaque. Accordingly, we performed simulations of the HT dimer at the identical EEF strengths with 0.1 GHz frequency that was performed for 10-peptides plaque and recorded their decomposition times based on the interpeptide distances, as shown in Figures S5.

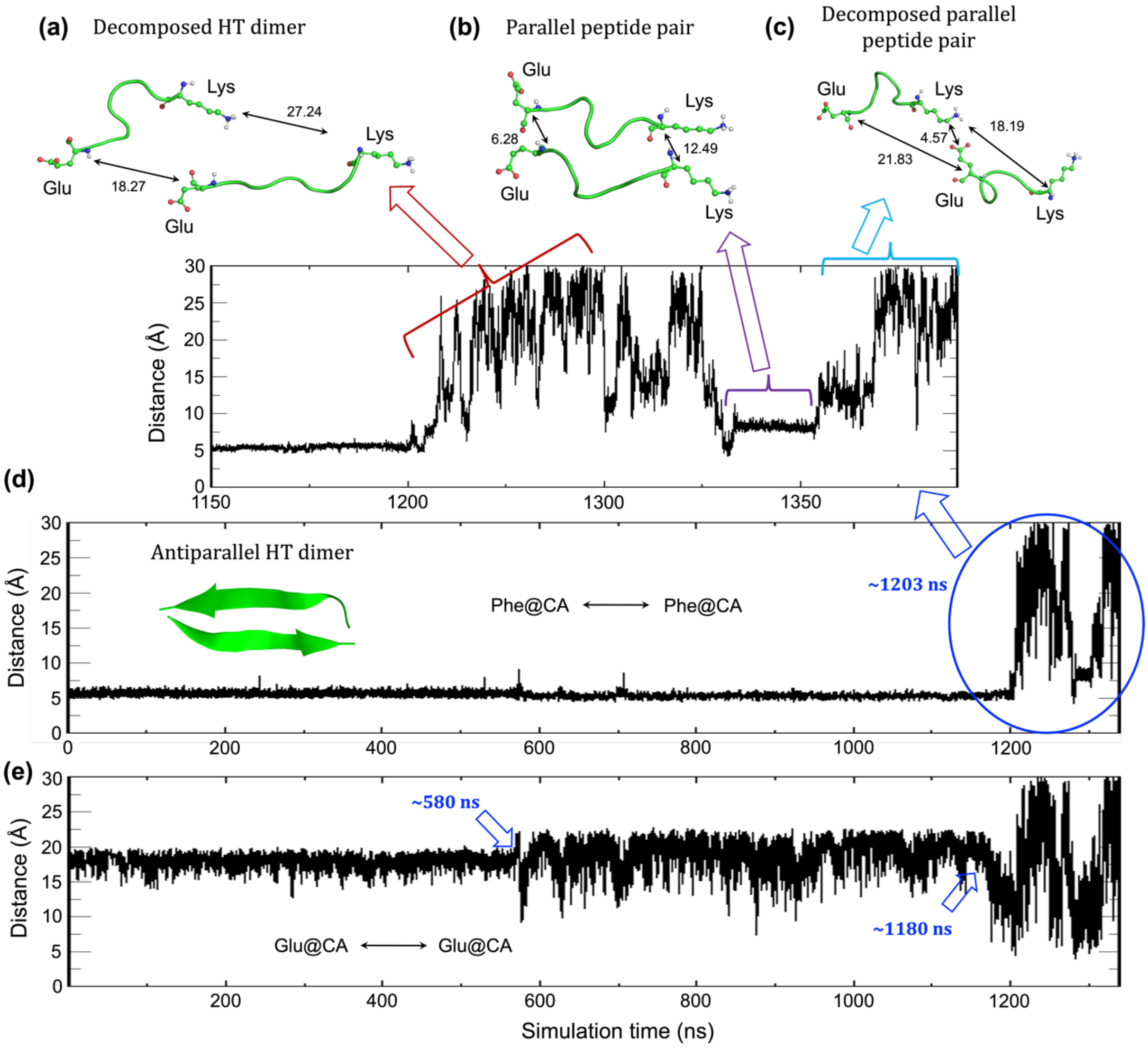

Figure S6: (a-c) Different conformations of the HT dimer obtained at various timescales during simulation. (d) The evolution of the interpeptide distance of the HT dimer at 3×10^{-6} V/Å. Similar to other simulations, the interpeptide distance was calculated by selecting the backbone carbon atoms of two phenylalanine residues, as shown in Figure 5a. Panel (e) shows the evolution of the interpeptide distance between the Glu-Glu residues.

We then correlated the decomposition times with the applied EEF strengths, as illustrated in Figure 12b (see main text). Similarly, the scattered data points were fitted with the logarithmic equation **y = −143.9 ln(x) −550.74**, which has an **R^2 value of 0.9659**. Using this equation, we predict that the timescale required for the separation of the HT dimer at **3×10^{-6} V/Å is approximately 1279 ns.** We then performed a simulation of the HT dimer at 3×10^{-6} V/Å coupled with a 0.1 GHz frequency and observed the separation of HT dimer **at approximately 1203 ns**, as shown in Figure S6. *The separation time of ~1203 ns is in good agreement with the predicted time of ~1279 ns.* This observation further validates that an experimentally viable EEF strength can separate the HT dimer. Therefore, we anticipate that a similar decomposition event should be possible for the 10-peptide plaque if we continue the simulation for a sufficiently long timescale.

A closer inspection of the HT dimer trajectory reveals several interesting events, which are better understood through the evolution of the interpeptide distance between the Glu-Glu residues. The Glu residue is located at the end of the 7-residue short peptide, and in the antiparallel arrangement, the two Glu residues on opposite strands are positioned at the maximum possible distance (~19 Å) from each other, as seen in Figure S6e. However, as the simulation progresses, around 580 ns, we observe the first significant conformational instability in the HT dimer. This causes the distance between the Glu-Glu residues to decrease and fluctuate significantly. Due to the continuous application of the EEF, the Glu-Glu residues attempt to move closer, and at approximately 1180 ns, there is a sudden drop in the Glu-Glu distance to approximately 6 Å. Nevertheless, the HT dimer is not yet completely separated, as indicated by the interpeptide distance measured by the backbone carbon atoms of two phenylalanine residues, shown in Figure S6d, which remains around 5-6 Å. However, the HT dimer then experiences severe instability, with weak intermolecular interactions unable to sustain the structure, leading to its immediate separation at approximately 1203 ns, as shown in Figures S6a and S6d. After continuing the simulation for an additional 100 ns, the EEF causes the separated peptides to come closer and start forming parallel pairs, as shown in Figure S6b. However, these parallel pairs are subsequently disrupted by the oscillatory effects of the field and separate again, as illustrated in Figure S6c. *This observation further supports our proposed idea of using Os-EEF with short pulses to efficiently decompose the plaque and prevent the formation of parallel pairs.* Furthermore, it is important to note that this effect is also pronounced at very low EEF strengths.

[**Note:** The simulation timescale obtained at different electric field strengths does not directly correlate with real-time duration. Additionally, a shorter timescale implies fewer sampled configurations relative to a longer one. A stronger field may enhance sampling efficiency by accelerating transitions between different conformations, whereas a weaker field may lead to slower sampling and longer simulation times required to observe rare events.]

S.5. Analysis of the Heating Effects

To understand the heating effects, we performed simulations by removing the thermostat once the system reached an equilibrium temperature of 300 K. We assessed the different heating effects under the following conditions: (a) with electric field (EF) and thermostat, (b) without EF and without thermostat, and (c) with EF but without thermostat.

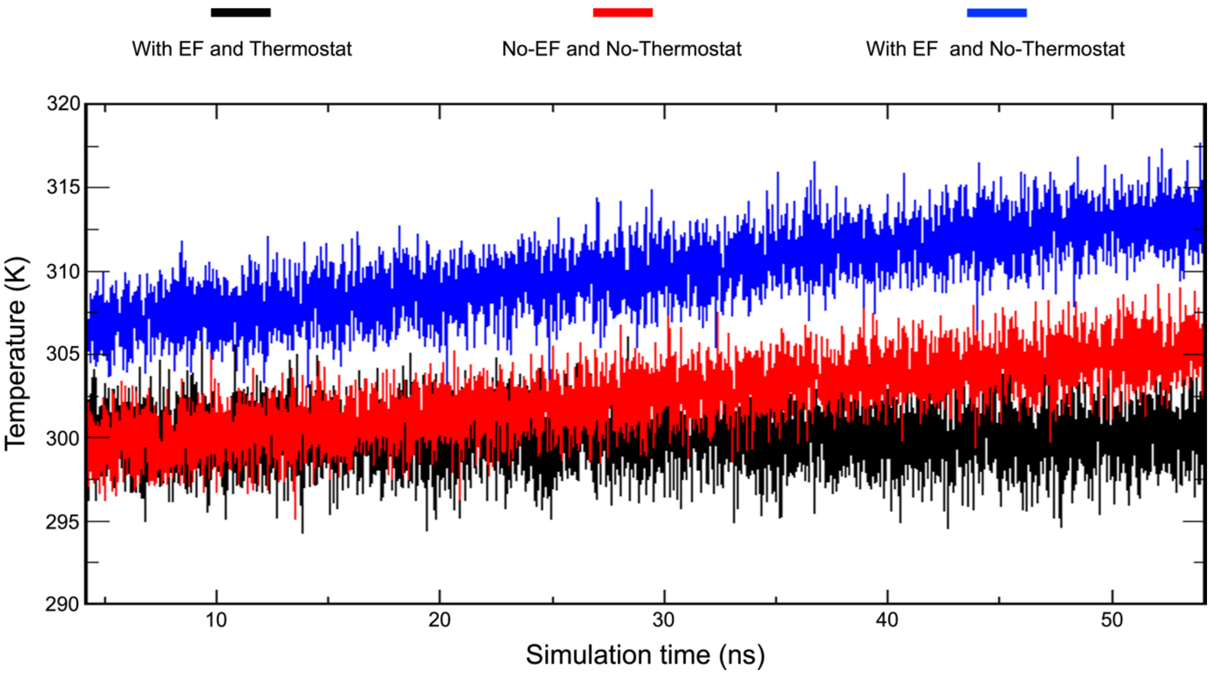

Figure S7: Evolution of temperature under different conditions, starting from 300 K.

As shown in Figure S7, the system's temperature gradually increases once the thermostat is removed, regardless of the presence or absence of the EEF. However, the rate of temperature increase is higher in the presence of EEF (see blue plot) compared to its absence (see red plot). The average temperature over the simulation time is 309.98 K with EEF (blue) and 302.45 K without EEF (red). In contrast, when both the EEF and thermostat are present (see black plot), the system temperature remains stable at an average of 300.04 K, which is the target temperature for our simulation. This stability demonstrates that the thermostat effectively manages the additional heat generated by the EEF. Therefore, we can conclude that the EEF

does not induce more heating than the thermostat can handle, indicating that the system does not overheat with the application of a 0.02 V/Å field strength.

S.6. Procedures for the Umbrella Sampling Simulations

Before conducting the umbrella sampling calculation, we first ensured the stability of the antiparallel β-sheet dimer by running an unbiased simulation for 50 ns. Subsequently, we performed umbrella sampling simulation to fully separate the antiparallelly bound β-strands, utilizing the center of mass (COM) restraint method (https://github.com/callumjd/AMBER-Umbrella_COM_restraint_tutorial). To achieve this, we needed to pull both strands apart using a force constant. We observed that in order to see a significant change in the separation of the peptides, we needed to create a substantial separation in their center of mass. Peptides are inherently flexible, so a minor change in the center of mass is usually balanced by slight adjustments in the peptide conformation, without causing significant changes in their separation.

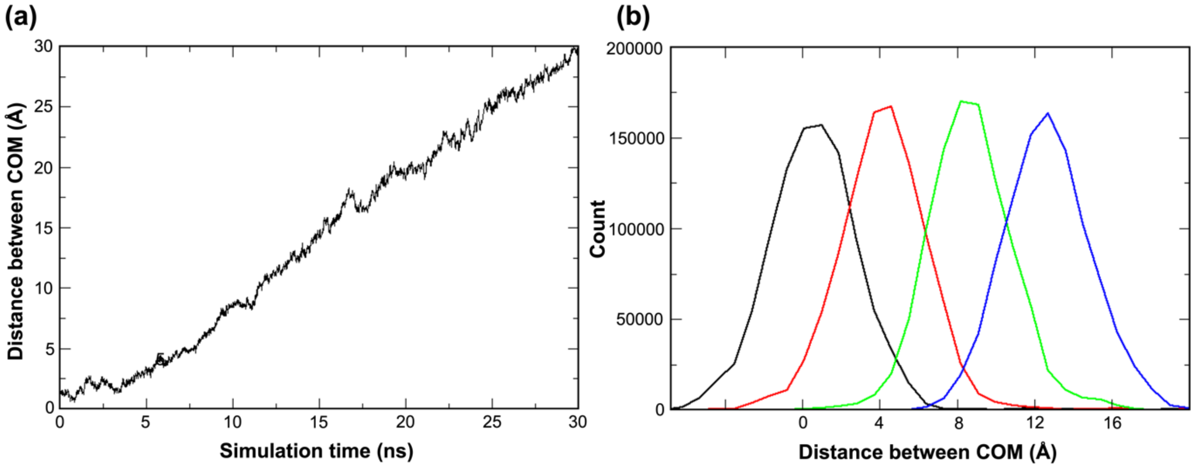

Figure S8: (a) Uniform evolution of distance between the COM of both peptides with progress of the simulation. (b) Depiction of significant overlap of windows simulated for umbrella sampling.

Therefore, we conducted a 30 ns simulation and pulled them apart employing the force constant of magnitude 1 kcal/(mol Å^2) by a distance of 30 Å, using the COM-COM distance as the chosen reaction coordinate. Figure S8a displays the uniform evolution of distance between the COM of both peptides with progress of the simulation. Following this, we generated windows based on the distance plot, each with a 4 Å COM separation, and conducted 5 ns simulations for each window. Note that we kept the identical parameters for all simulations as mentioned in S.2. The selection of the number of windows was determined by the interactions between the strands. Once the interactions between the strands reduced to zero, we

ceased simulating additional windows. To further validate our approach, Figure S8b demonstrates the significant overlap between the windows, which is a crucial measure to confirm the accuracy of the umbrella sampling calculations. The PMF was generated using the WHAM software (http://membrane.urmc.rochester.edu/?page_id=126).

S.7. Alternative Descriptions of the Rapid Decomposition of Plaques

An alternative representation of the plaque decomposition process is displayed in Figure S9, which illustrates the root-mean-square deviation (RMSD) of the peptide backbone atoms and the evolution of the number of hydrogen bonds over time. The RMSD plot, in Figure S9a, exhibits a jump within 50 ns, and it rises to 15 to 17 Å. This rapid rise in the RMSD within 50 ns, signifies the onset of a major instability in the plaque, resulting in an instant separation to the 10 individual peptides. Thereafter, the RMSD shows fluctuations around an average value of 17 Å.

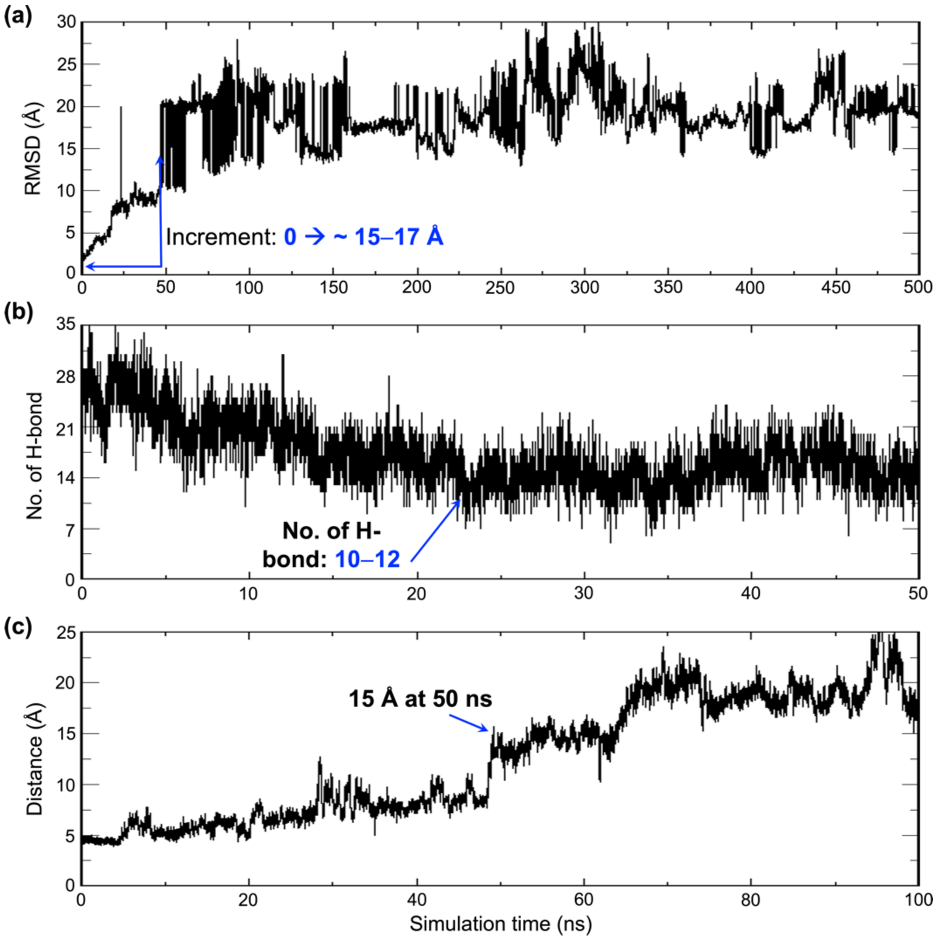

Figure S9: (a) The RMSD plot highlights the acquired instability in the plaque during the initial 50 ns timescale for the Os-EEF with frequency 0.1 GHz and strength 0.02 V/Å. (b) Evolution of the number of hydrogen bonds for the first 50 ns of simulation time. (c) Evolution of the interpeptide distance (in Å) with the progress of the simulation for a replica simulation in the identical condition focusing on the backbone carbon atoms, as shown in Figure 1a.

The rapid decomposition of the plaque can also be deduced from Figure S9b. As such, the number of hydrogen bonds, a measure of secondary structure content in peptides, significantly decreases from ~30 to ~10-12 within the initial 25 ns, indicating the rapid disruption of the secondary β-sheet structure, which is the primary building block of mature plaques. Additional evidence is found in the evolution of secondary structure over time, revealing the absence of secondary β-sheet at around 50 ns, as shown in Figure S10.

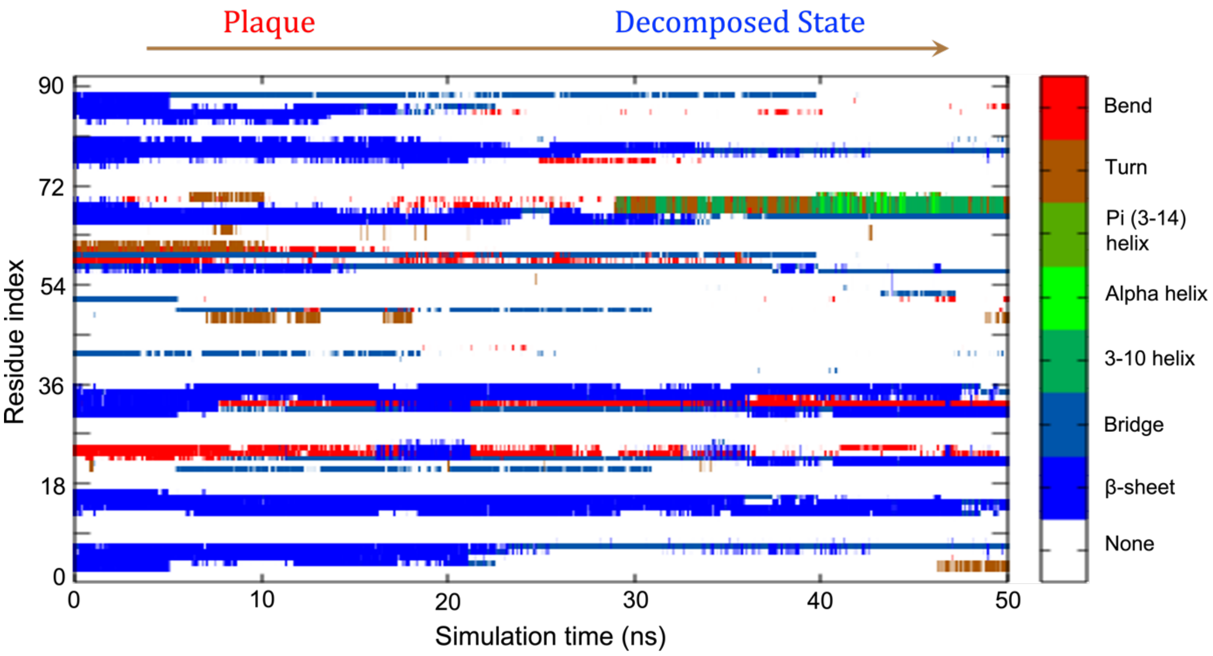

Figure S10: Decay of the secondary structure with progress of the simulation at 0.1 GHz frequency with EEF strength 0.02 V/Å. Each 9-residue index refers to a single peptide chain.

S.8. Periodic Buildup and Destruction of Parallel Pairs

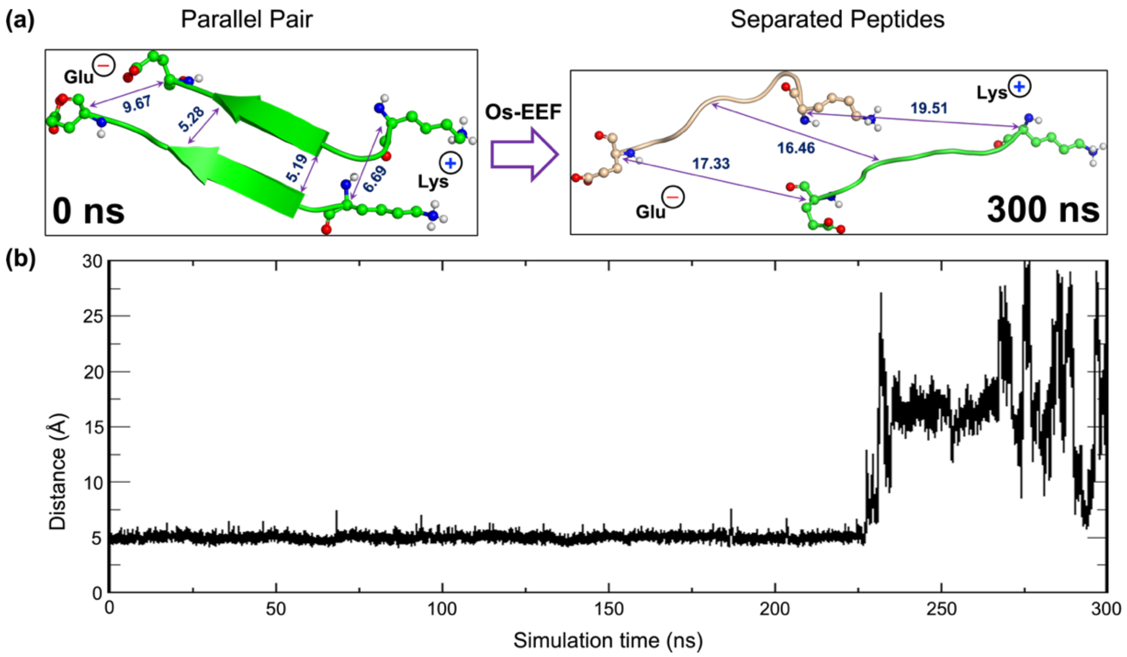

Figure S11: (a) Formation and destruction of a parallel-pair (**PP**) structure, which is formed due to a prolonged exposure to Os-EEF (0.01 GHz with 0.02 V/Å) after the plaque explosion. The initial coordinates at 0 ns were taken from Figure 6b. All double-headed arrows indicate interpeptide backbone-backbone distances in Å. (b) Evolution of interpeptide distance within the **PP** during the simulation. The separation is calculated using the backbone carbon atoms of a phenylalanine pair.

As shown in Figure S11a, the interpeptide distance within the parallel pair (PP) is initially around 5 Å but increases to 17-18 Å as the simulation progresses. This dispersion is supported by the evolution of interpeptide distance shown in Figure S11b. It reveals an increasing separation after ~230 ns and marks the complete destruction of the **PP**s by Os-EEF (0.1 GHz with 0.02 V/Å). The corresponding RMSD plot (see Figure S12) of the backbone atoms substantiates the PP separation and reveals a sudden instability after 230 ns.

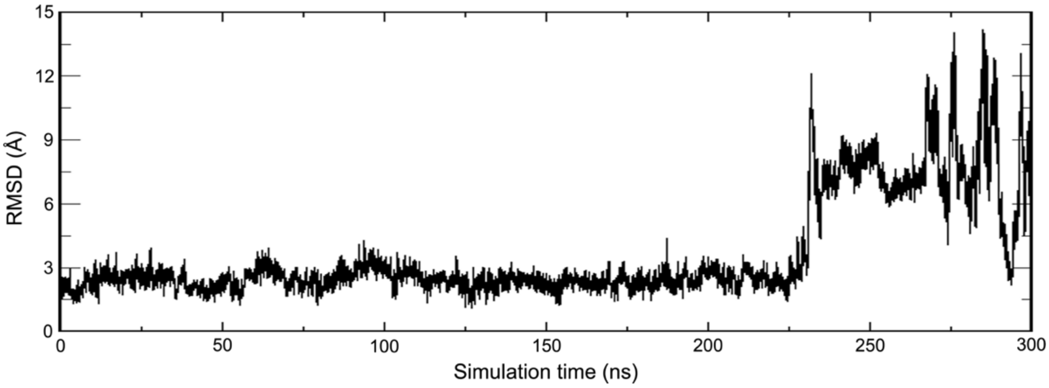

Figure S12: RMSD plot for the simulation of parallel pair under Os-EEF (0.01 GHz with 0.02 V/Å), showing the sudden instability after around 230 ns.

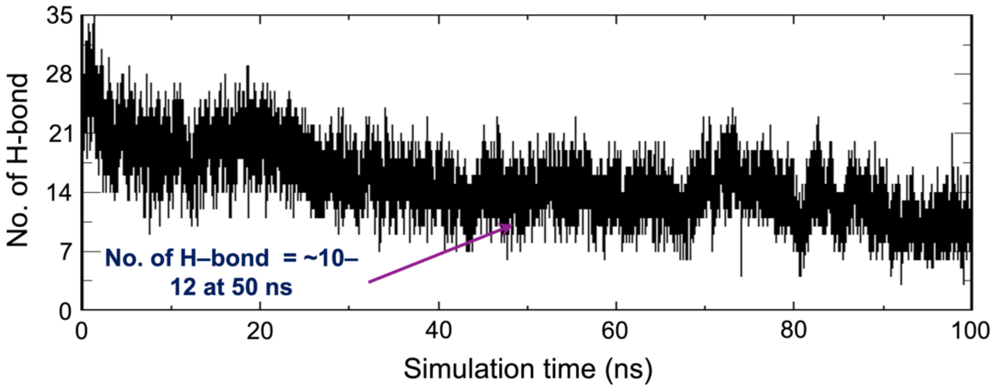

Figure S13: Evolution of the number of hydrogen bonds with progress of the simulation in the presence of Os-EEF at 0.02 V/Å and a frequency of 20 MHz.

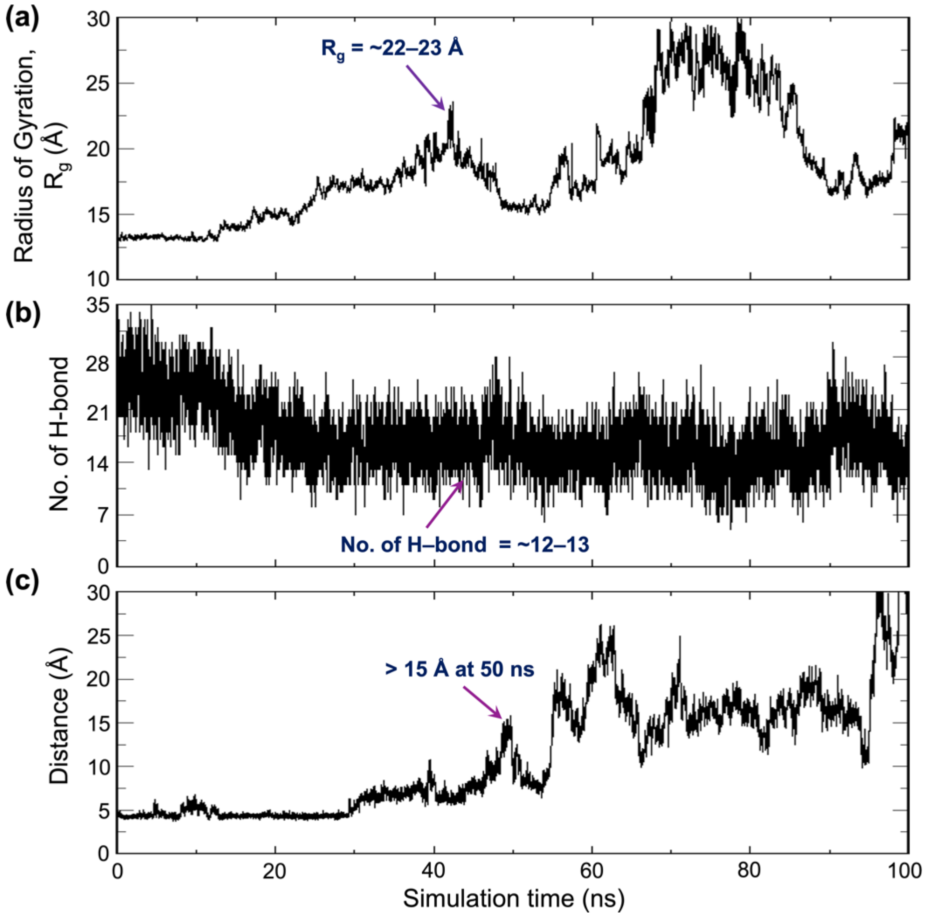

Figure S14: The evolution of (a) the radius of gyration and (b) the number of hydrogen bonds in 10-peptide plaques as the simulation progresses, under the influence of an Os-EEF of 0.02 V/Å with a frequency of 20 MHz. (c) Evolution of the interpeptide distance (in Å) with the progress of the simulation in the identical condition focusing on the backbone carbon atoms, as shown in Figure 1a.

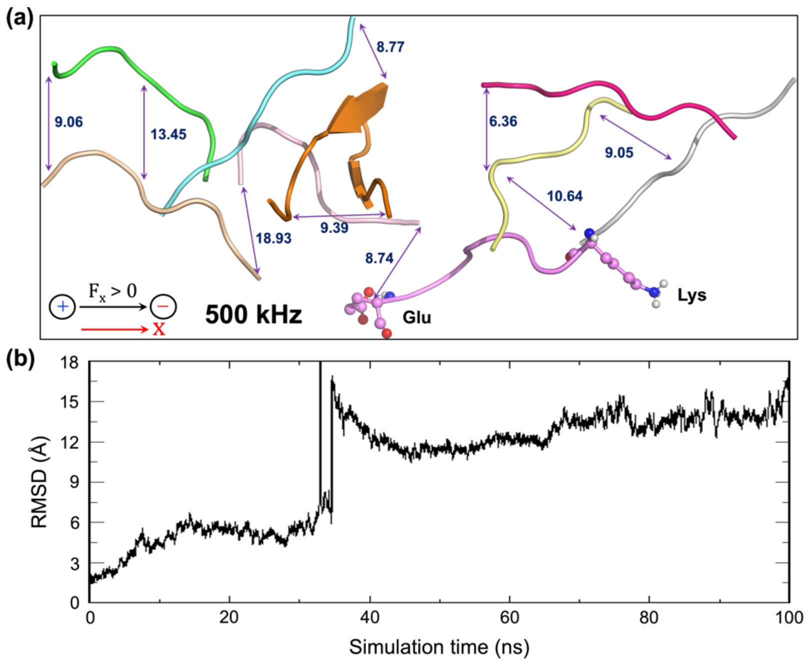

Figure S15: (a) The decomposition of a 10-peptide plaque at 50 ns, due to exposure to an Os-EEF (along the x-axis) of 0.02 V/Å with a frequency of 500 kHz. The double-headed arrows indicate several representative interpeptide distances (in Å) among the dispersed peptides. The black arrow in the top right corner marks the direction of the applied Os-EEF. (b) The corresponding RMSD of backbone atoms over the course of the simulation.

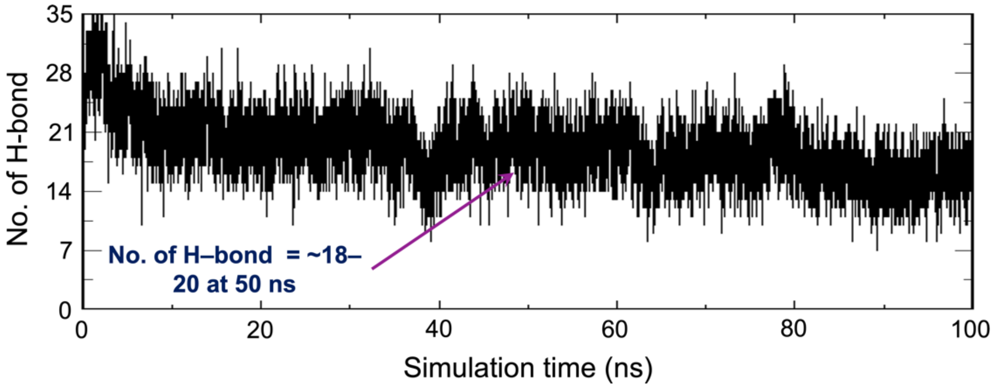

Figure S16: Evolution of the number of hydrogen bonds with progress of the simulation in the presence of Os-EEF at 0.02 V/Å and a frequency of 1 THz.

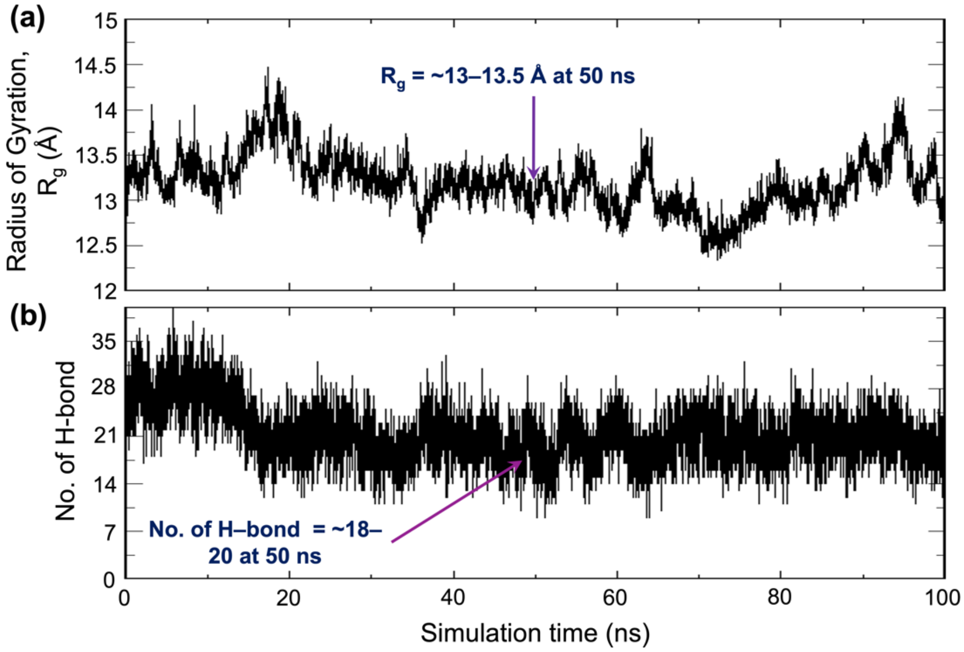

Figure S17: The evolution of (a) the radius of gyration and (b) the number of hydrogen bonds in 10-peptide plaques as the simulation progresses, under the influence of an Os-EEF of 0.02 V/Å with a frequency of 1 THz.

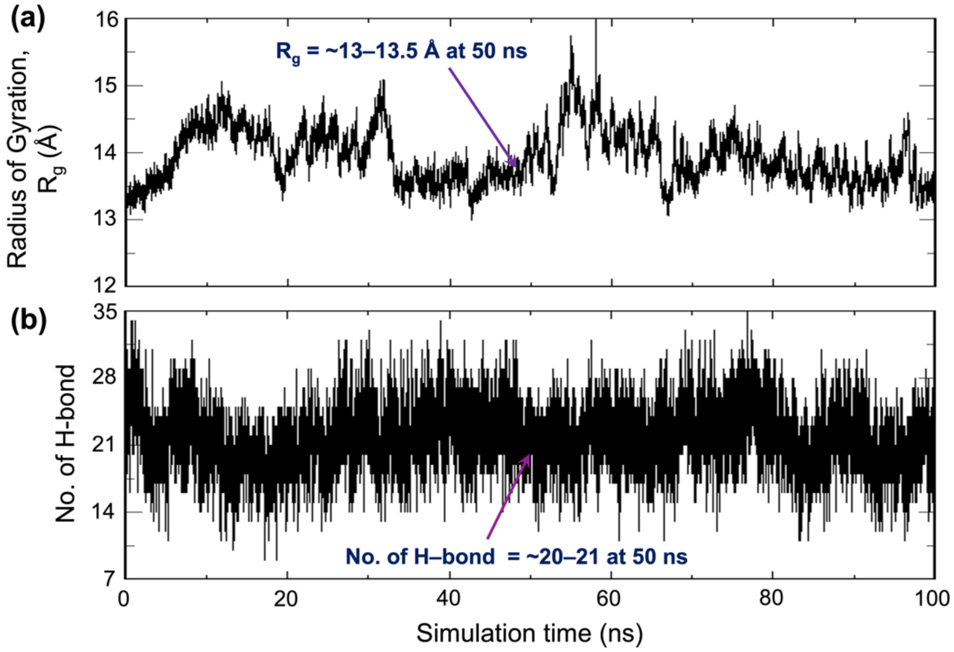

Figure S18: The evolution of (a) the radius of gyration and (b) the number of hydrogen bonds in 10-peptide plaques as the simulation progresses, under the influence of an Os-EEF of 0.02 V/Å with a frequency of 10 THz.

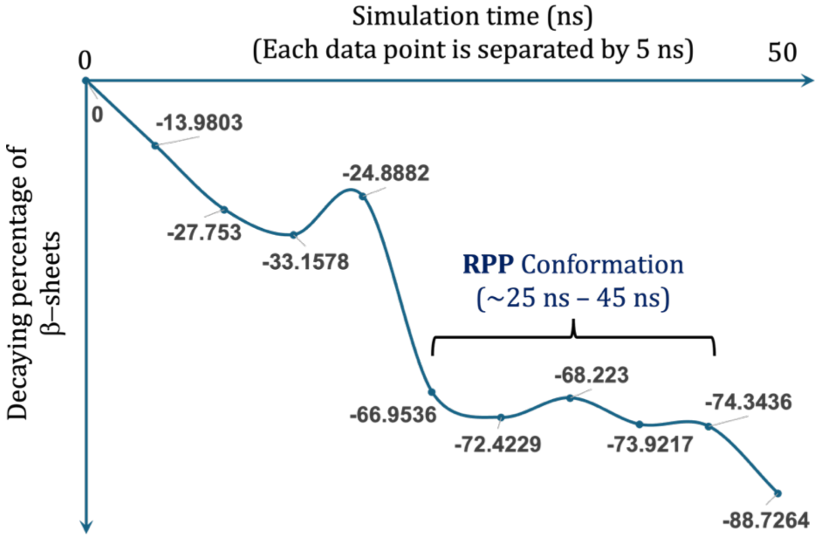

Figure S19: Percentage of β-sheets decay in the presence of Os-EEF with frequency 0.1 GHz at a strength of 0.02 V/Å. Each data point represents the change in β-sheet percentage relative to the initial value observed in the amyloid plaque at 0 ns.

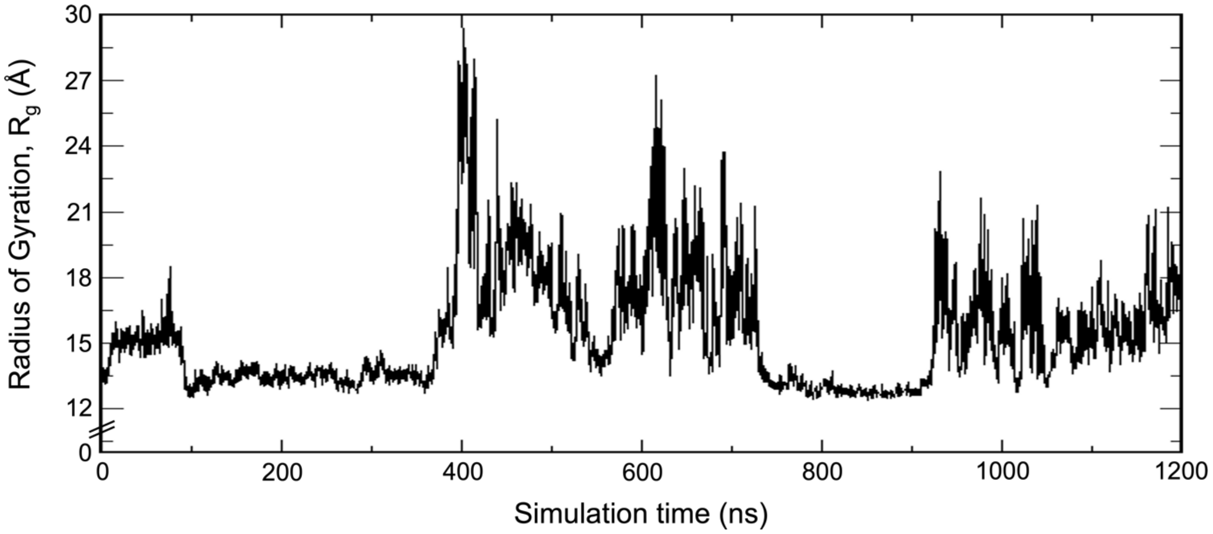

Figure S20: The evolution of the radius of gyration of 10-peptide plaques as the simulation progresses, in the presence of an St-EEF with a strength of 0.02 V/Å.

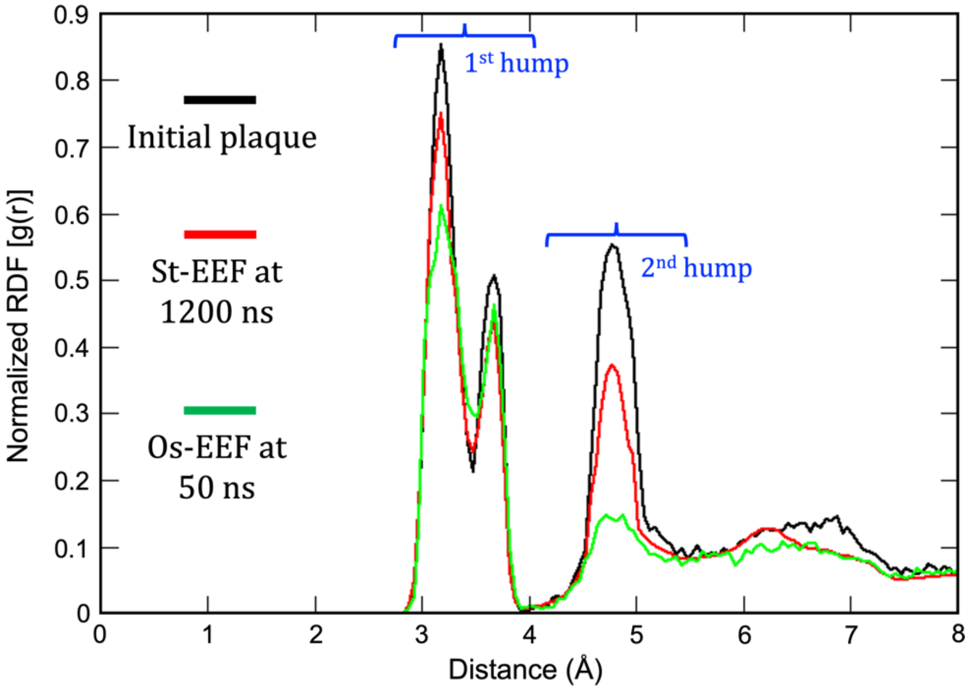

Figure S21: Peptide-peptide radial distribution function (RDF) plot of the initial plaque and its decomposition by St-EEF as well as Os-EEF. Calculations are done using the AMBER inbuilt algorithm, focusing specifically on the backbone carbon atoms and utilizing data from the entire trajectory Note that the first hump primarily reflects the intrapeptide separation, originating from the interactions between backbone carbon atoms within the same peptide. The second hump corresponds to the interpeptide separation, indicating the distances between peptides. Notably, the initial plaque (black curve) exhibits the highest RDF, followed by the St-EEF (red curve), and the Os-EEF simulation (green curve) displays the lowest RDF. As the Os-EEF RDF is the lowest, it suggests greater peptide-peptide separation compared to St-EEF.

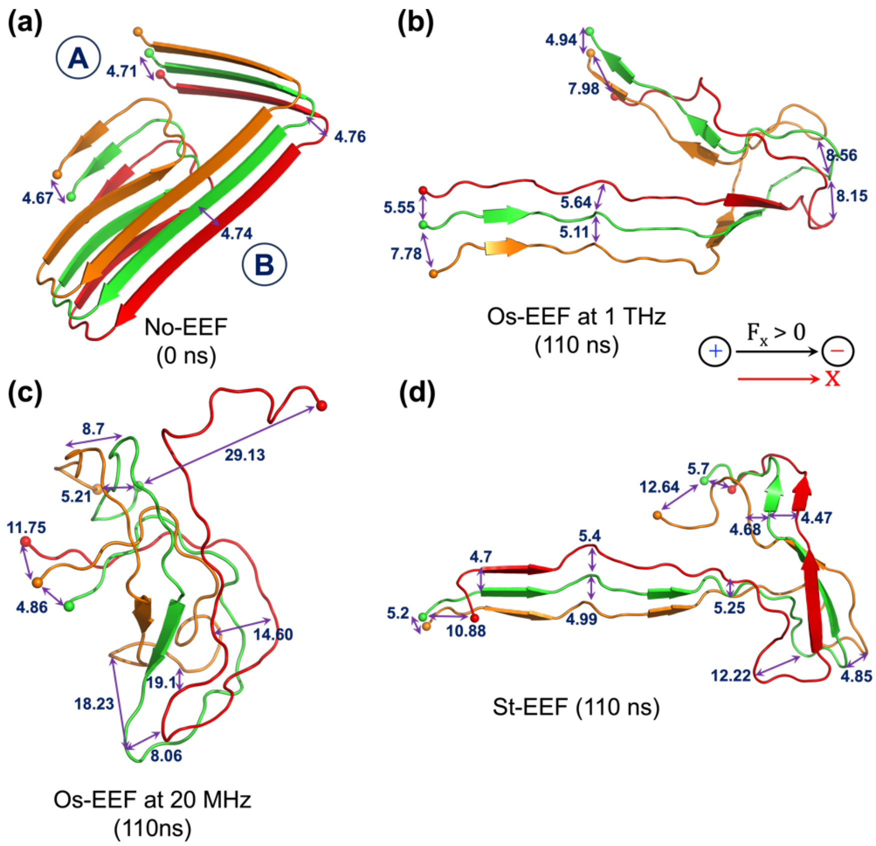

Figure S22: The conformations of the Aβ-42 trimer are shown as follows: (a) at 0 ns in the absence of EEF, (b) and (c) at 110 ns with Os-EEF applied at 1 THz and 20 MHz, respectively, and (d) at 110 ns with St-EEF. All conditions have an identical EEF strength of 0.02 V/Å. The double-headed arrows indicate the representative interpeptide distances in Å between the peptides.

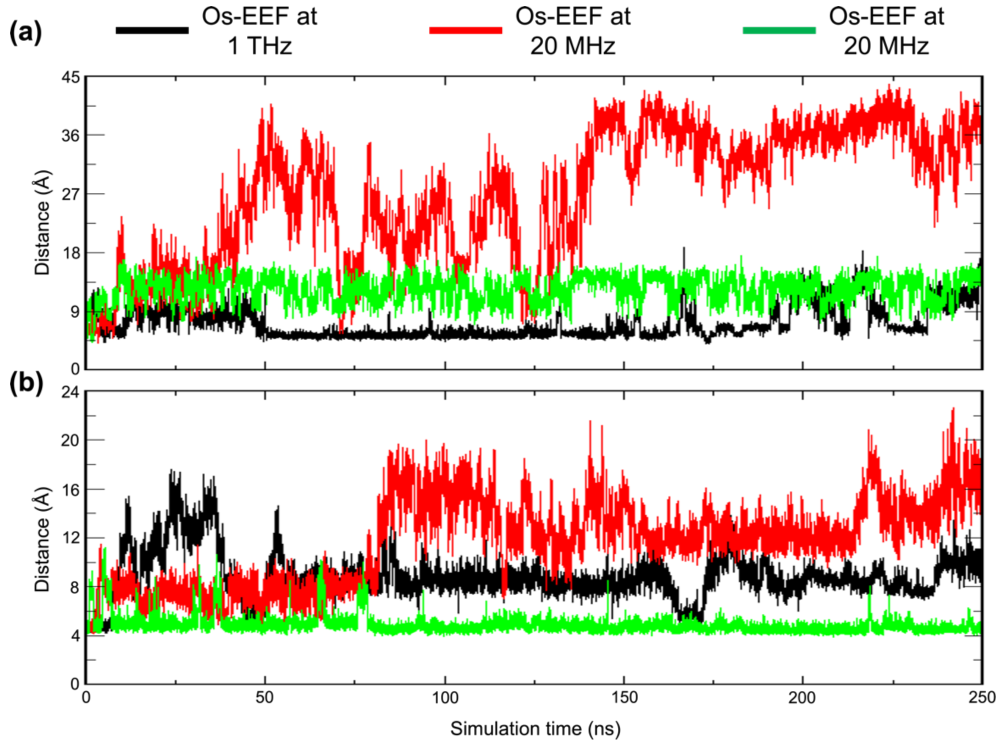

Figure S23: The evolution of interpeptide distances observed under different conditions is illustrated as follows: (a) represents the terminal-terminal distances, indicated by point (A) in Figure S22a, while (b) shows the non-terminal interpeptide distances, marked by point (B) in Figure S22a.

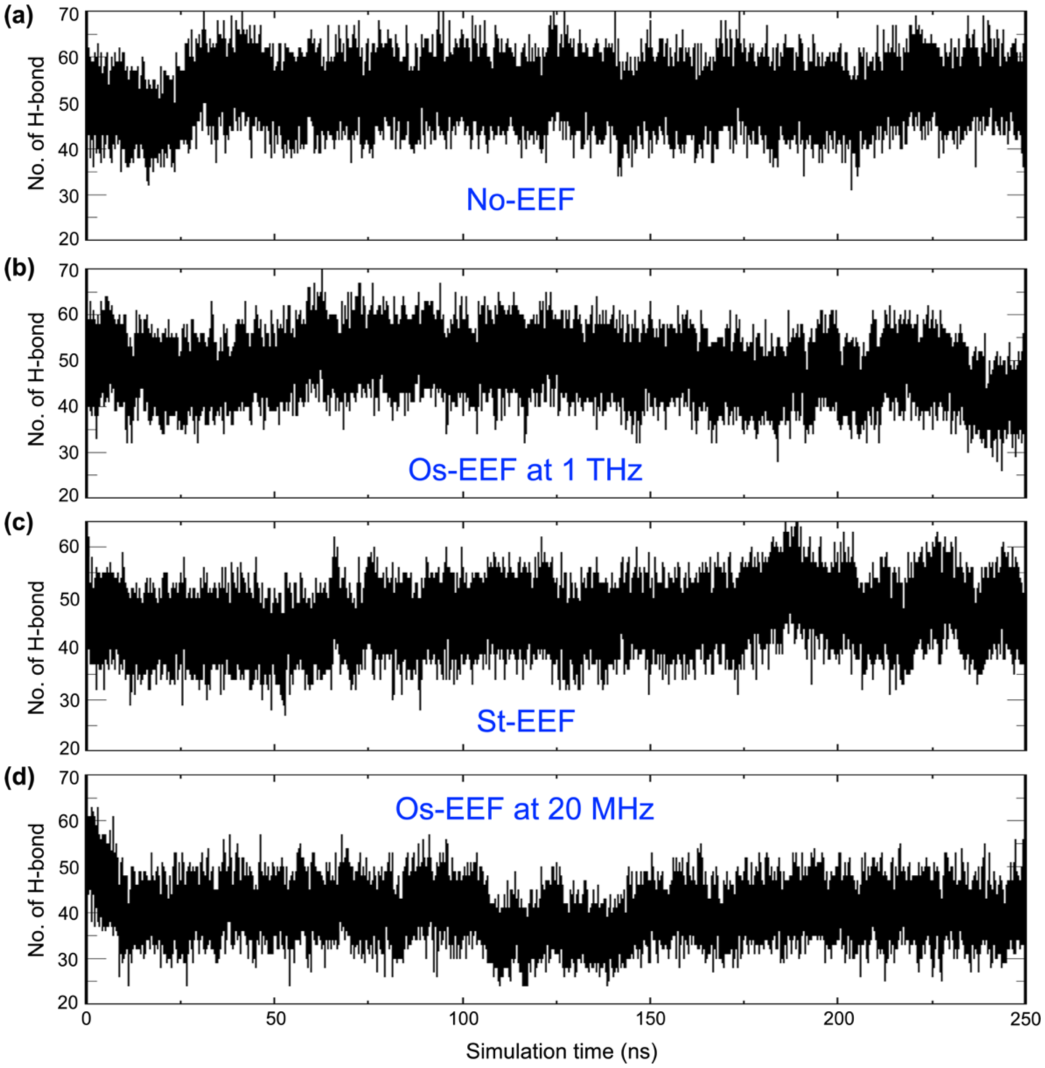

Figure S24: Evolution of the number of hydrogen bonds throughout the simulation under various conditions: (a) No-EEF, (b) Os-EEF with a frequency of 1 THz, (c) St-EEF, and (d) Os-EEF with a frequency of 20 MHz. In all cases, the EEF strength is 0.02 V/Å.

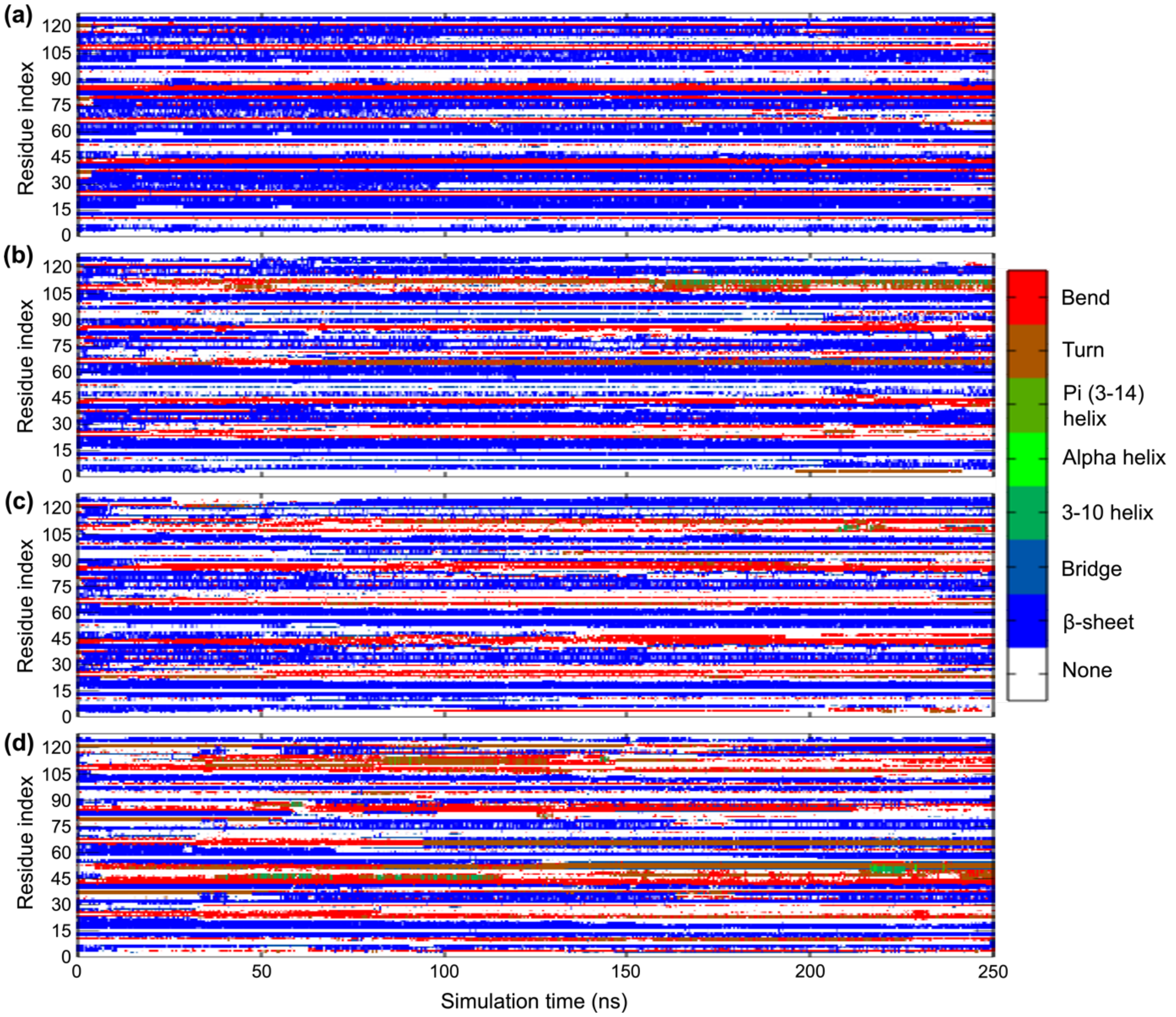

Figure S25: Decay of the secondary structure throughout the simulation under various conditions: (a) No-EEF, (b) Os-EEF with a frequency of 1 THz, (c) St-EEF, and (d) Os-EEF with a frequency of 20 MHz. The plots were generated from the trajectory using the AMBER-built CPPTRAJ program with the DSSP algorithm for secondary structure analysis.

Python Code for Velocity Calculation:

```
from Bio.PDB import PDBParser
import numpy as np

# Atomic masses in amu
atomic_masses = {
    "C": 12.011,
    "H": 1.008,
    "N": 14.007,
    "O": 15.999,
    "S": 32.060,
}

# Function to calculate COM
```

```
def calculate_com(coordinates, masses):
    com_x = np.sum(masses * coordinates[:, 0]) / np.sum(masses)
    com_y = np.sum(masses * coordinates[:, 1]) / np.sum(masses)
    com_z = np.sum(masses * coordinates[:, 2]) / np.sum(masses)
    return com_x, com_y, com_z

parser = PDBParser(QUIET=True)

# Paths to PDB files
initial_pdb = "initial.pdb"
final_pdb = "final.pdb"

# Parsing the PDB files
initial_struc = parser.get_structure("initial", initial_pdb)
final_struc = parser.get_structure("final", final_pdb)

# Lists to store coordinates and masses at initial and final steps
coordinates_initial = []
masses_initial = []
coordinates_final = []
masses_final = []

# Reading atoms for initial
for atom in initial_struc.get_atoms():
    # Extract the atomic symbol from the last column
    atomic_symbol = atom.get_id()[0].strip()
    if atomic_symbol in atomic_masses:
        coordinates_initial.append(atom.coord)
        masses_initial.append(atomic_masses[atomic_symbol])

# Reading atoms for final structure
for atom in final_struc.get_atoms():
    atomic_symbol = atom.get_id()[0].strip()
    if atomic_symbol in atomic_masses:
        coordinates_final.append(atom.coord)
        masses_final.append(atomic_masses[atomic_symbol])

# Converting lists to arrays
coordinates_initial = np.array(coordinates_initial)
masses_initial = np.array(masses_initial)
coordinates_final = np.array(coordinates_final)
masses_final = np.array(masses_final)

# Calculate COM at initial and final
com_x_initial, com_y_initial, com_z_initial = calculate_com(coordinates_initial, masses_initial)
com_x_final, com_y_final, com_z_final = calculate_com(coordinates_final, masses_final)

# Displacement of the COM
displacement_x = com_x_final - com_x_initial
```

```
displacement_y = com_y_final - com_y_initial
displacement_z = com_z_final - com_z_initial

# Magnitude of displacement
displacement_magnitude = np.sqrt(displacement_x**2 + displacement_y**2 + displacement_z**2)

# Print the displacement vector and magnitude
print(f"Displacement Vector (x, y, z): ({displacement_x:.4f}, {displacement_y:.4f}, {displacement_z:.4f}) Angstroms")
print(f"Displacement Magnitude: {displacement_magnitude:.4f} Angstroms")

# Velocity calculation
time = xy #(in ps)
velocity = displacement_magnitude / time  # Angstrom/ps
print(f"Velocity: {velocity:.4f} Angstroms/ps")
```

References:

1. Izaguirre, J. A.; Catarello, D. P.; Wozniak, J. M.; Skeel, R. D. Langevin stabilization of molecular dynamics. *J. Chem. Phys.* **2001**, *114*, 2090−2098.

2. Berendsen, H. J. C.; Postma, J. P. M.; van Gunsteren, W. F.; DiNola, A.; Haak, J. R. Molecular dynamics with coupling to an external bath. *J. Chem. Phys.* **1984**, *81*, 3684−3690.

3. Åqvist, J.; Wennerström, P.; Nervall, M.; Bjelic, S.; Brandsdal, B. O. Molecular dynamics simulations of water and biomolecules with a Monte Carlo constant pressure algorithm. *Chem. Phys. Lett.* **2004**, *384*, 288−294.

4. Ryckaert, J.-P.; Ciccotti, G.; Berendsen, H. J. C. Numerical integration of the cartesian equations of motion of a system with constraints molecular dynamics of n-alkanes. *J. Comput. Phys.* **1977**, *23*, 327−341.

5. Darden, T.; York, D.; Pedersen, L. Particle mesh Ewald: an N.log(N) method for Ewald sums in large systems. *J. Chem. Phys.* **1993**, *98*, 10089−10092.
